# Proteomic Approach for Searching for Universal, Tissue-Specific, and Line-Specific Markers of Extracellular Vesicles in Lung and Colorectal Adenocarcinoma Cell Lines

**DOI:** 10.3390/ijms21186601

**Published:** 2020-09-09

**Authors:** Svetlana Novikova, Natalia Shushkova, Tatiana Farafonova, Olga Tikhonova, Roman Kamyshinsky, Victor Zgoda

**Affiliations:** 1 Orekhovich Institute of Biomedical Chemistry of Russian Academy of Medical Sciences, Pogodinskaya 10, 119121 Moscow, Russia; farafonova.tatiana@gmail.com (T.F.); tiolika@gmail.com (O.T.); victor.zgoda@gmail.com (V.Z.); 2 National Research Center “Kurchatov Institute”, Akademika Kurchatova pl. 1, 123182 Moscow, Russia; kamyshinsky.roman@gmail.com; 3 Shubnikov Institute of Crystallography of Federal Scientific Research Centre ‘Crystallography and Photonics’ of Russian Academy of Sciences, Leninskiy Prospect, 59, 119333 Moscow, Russia; 4 Moscow Institute of Physics and Technology, Institutsky Lane 9, Dolgoprudny, 141700 Moscow, Russia

**Keywords:** extracellular vesicles, exosomes, proteomics, mass-spectrometry, colon cancer, lung cancer, biomarkers

## Abstract

Tumor-derived extracellular vesicles (EVs), including exosomes, contain proteins that mirror the molecular landscape of producer cells. Being potentially detectible in biological fluids, EVs are of great interest for the screening of cancer biomarkers. To reveal universal, tissue-specific, and line-specific markers, we performed label-free mass spectrometric profiling of EVs originating from the human colon cancer cell lines Caco-2, HT29, and HCT-116, as well as from the lung cancer cell lines NCI-H23 and A549. A total of 651 proteins was identified in the EV samples using at least two peptides. These proteins were highly enriched in exosome markers. We found 11 universal, eight tissue-specific, and 29 line-specific markers, the levels of which were increased in EVs compared to the whole lysates. The EV proteins were involved in the EGFR, Rap1, integrin, and microRNA signaling associated with metastasis and cancer progression. An EV protein-based assay could be developed as a liquid biopsy tool.

## 1. Introduction

According to the World Health Organization (WHO) data for 2018, lung cancer (LC) and colorectal cancer (CRC) are the most common causes of cancer-related deaths worldwide, accounting for an estimated 1.76 million and 862,000 deaths, respectively. They also rank first (2.09 million cases) and third (1.80 million cases) in terms of occurrence (https://www.who.int/news-room/fact-sheets/detail/cancer).

For LC, non-small cell lung carcinoma (NSCLC) represents 85% of all primary lung tumor cases. NSCLC generally encompasses adenocarcinoma (38.5% of all cases), squamous cell carcinoma (20% of all cases), and large cell carcinoma (2.9% of all cases) [1]. In recent years, the incidence of adenocarcinoma has increased and has overtaken squamous cell carcinoma in terms of prevalence [1,2]. Only 15% of patients with NSCLC are diagnosed at an early stage, while late diagnosis and metastatic disease are associated with a five-year survival rate of 3.9% [3]. Screening imaging techniques, i.e., low-dose computed tomography (LDCT), reduce mortality by 20% in high-risk patients with a history of smoking [3]. However, imaging techniques have the limitations of a high-false positive rate, over diagnosis, and increased radiation exposure [4]. Once a pulmonary lesion is detected by the imaging method, it is mandatory to perform a biopsy. Computed tomography (CT)-guided lung biopsy and flexible bronchoscopy are the most common diagnostic procedures [5]. Their main complications include pneumothorax (20–25% of cases) and pulmonary hemorrhaging (41.1%), which in rare cases can be severe. Moreover, a potentially lethal air embolism occurs in 0.061% of cases. [5]. Nevertheless, depending on the patient’s characteristics, less invasive but less sensitive methods of LC diagnosis (e.g., sputum cytology) are preferred.

For CRC, 95% of all colorectal tumors are adenocarcinomas, but they differ internally in their molecular characteristics. Overall, 70–90% of colorectal cancers are associated with the chromosomal instability pathway (CIN), while 10–20% of colorectal cancers represent the serrated neoplasia pathway characterized by the CpG island methylation phenotype (CIMP) [6]. For advanced tumors, the three-year survival rate is 16%. Screening for CRC is performed by means of stool-based tests followed by a colonoscopy if positive. Complications occur in 1% of cases and include post-polypectomy bleeding, as well as perforation [7].

Conventional biopsy consists of histological (visual identification of cancerous cells), immunohistochemical (expression of TTF1, p40 or p63, CK5/6, napsin or CK7, PD-L1 (LC) and MLH1, MSH2, MSH6, and PMS2 (CRC)), and genetic (search for mutations in *EGFR*, *ALK*, *ROS-1* genes (LC) and in *KRAS*, *NRAS, BRAF, PIK3CA, PTEN* genes (CRC); CIN and CIMP status, microsatellite instability (MSI) status (CRC) associated with the response to targeted therapy) analyses of a tissue sample obtained by an invasive method [8,9].

Liquid biopsy or blood specimen analyses are alternative or complementary approaches to traditional imaging screening followed by biopsy for the diagnosis of LC and CRC [10,11]. This approach is useful when the amount of tissue is insufficient for convenient molecular testing, i.e., immunohistochemistry (IHC) or fluorescence in situ hybridization (FISH), or if a traditional biopsy would be associated with high risk due to the lesion localization. Liquid biopsy can be applied for cancer screening, searching for prognostic and predictive markers, assessing residual disease, and patient follow-up [10]. Liquid biopsy targets circulating tumor DNA (ctDNA), tumor-associated antigens, tumor educated platelets, tumor-associated autoantibodies, circulating tumor cells (CTCs), micro RNA (miRNA), and extracellular vesicles (EVs). However, additional clinical trials, rigorous standardization, and increased sensitivity (e.g., for CTCs detection) are needed for the liquid biopsy to become the diagnostic method of first choice rather than a complementary test.

EVs represent appealing potential biomarkers. The term “extracellular vesicles” encompasses the highly heterogenetic population of secreted membrane vesicles that differ by their size and molecular cargo [12]. EVs are secreted by all types of cells and can be found in all types of bodily fluids, including the blood (plasma and serum) [13]. Based on the mechanism of biogenesis, two large classes, i.e., exosomes (30–100 nm in diameter) and microvesicles (50 to 1000 nm in diameter), can be distinguished [12]. It is generally thought that exosomes develop from endosomes whose membranes bud inward and form intraluminal vesicles (ILVs). This process underlies endosome maturation. Endosomes with ILVs inside are considered multivesicular endosomes (MVEs) secreted through the fusion of MVEs with the plasma membrane [13]. The mechanism for the formation of MVBs involves the endosomal sorting complex required for transport (ESCRT), whose components HGS, TSG101, VPS4, VPS32, and PDCD6IP (ALIX) serve as molecular hallmarks of exosomes [12,13]. Moreover, a number of molecules, e.g., syntenin (SDCBP), syndecan (SDC4), and lactadherin (MFGE8); the lipid raft proteins FLOT1 and FLOT2, ARF6, VAMP3, and HSPA8; and tetraspanins CD9, CD63, CD81, and CD82 are considered exosome-specific proteins [13]. In turn, microvesicles are generated by the outward budding and fission of the plasma membrane. Microvesicle biogenesis induces changes in the lipid components, protein composition, and Ca^2+^ levels, and involves molecules such as aminophospholipid translocases (flippases and floppases), scramblases, and calpain [12]. The EVs internalize by recipient cells through membrane fusion, micropinocytosis, phagocytosis, receptor-mediated endocytosis involving caveolin, clathrin, lectins, adhesion molecules (e.g., ICAM1, MFGE8, integrins, etc.), and heparan sulfate proteoglycans (HSPGs) [14].

Functionally, EVs are part of a sophisticated transport network through which cells exchange messages with each other via the transfer of DNA, RNA, proteins, membrane-bound factors, etc. The molecular cargo of EVs can directly affect the tumor microenvironment or spread through the blood and lymph to distant sites. The latter method allows for the creation of a pre-metastatic niche—the so-called tumor macroenvironment—that promotes metastasis [15]. The mechanisms of cancer expansion via EVs are various and include immunosuppression, metabolic reprogramming, and the transfer of active oncogenes, e.g., EGFR, the mutant form of KRAS, and integrins into the cells of the target organ [16,17,18,19]. Taking into consideration the powerful and versatile contribution of EVs to metastasis, their molecular composition, especially the proteome, is of great interest.

EVs mirror the molecular features of parent tumor cells and preserve their molecular cargo (RNA, DNA, and proteins) from degradation via a bilayer lipid membrane [13]. In this context, tumor-derived EVs are a promising source of cancer biomarkers that can be detected in blood specimens, i.e., liquid biopsy, as mentioned above. In LC, exosomal miR-23a was shown to promote disease progression, and the surface proteins CD91, CD317, and EGFR were considered as LC diagnostic markers [11]. For CRC, it was shown that decreased levels of miR-638 in serum exosomes are associated with an increased risk metastasis [20]. Notably, liquid biopsies of LC and CRC are mainly focused on ctDNA, CTCs, or miRNA, while cancer-associated EV proteins are less commonly studied.

Recently, we reviewed the contribution of omics techniques, including mass-spectrometry, for the investigation of EV composition and the benefits of such an approach for clinical application [21]. High resolution shotgun mass-spectrometry provides a tool for the accurate identification and quantification of thousands of proteins [22]. To date, the liquid chromatography–tandem mass spectrometry (LC–MS/MS) analysis of HTC116-derived and HT29-derived small EVs revealed that TSPAN1 is a potent non-invasive biomarker for CRC detection [23]. The proteomic profiling of CRC SW620 cell-derived exosomes showed the enrichment of metastatic factors (MET, S100A8, S100A9, and TNC), signal transduction molecules (EFNB2, JAG1, SRC, and TNIK), and lipid raft and lipid raft-associated components (CAV1, FLOT1, FLOT2, and PROM1) compared with the nonmetastatic primary cell line, SW480 [24]. The mass-spectrometric profiling of NSCLC blood-sample-derived EVs and A549 cell line-derived EVs allows for the determination of fibronectin (FN1) as an LC marker [25]. Applying the triple SILAC quantitative proteomic strategy, a high abundance of EGFR, GRB2, and SRC was observed in the A549 and HCC827 line-derived EVs compared with the immortalized normal human bronchial epithelial cell line [26]. Despite these promising results, the proteomic composition of LC-derived and CRC-derived EVs needs to be further studied.

As mentioned above, adenocarcinoma represents the most common histological type of NSCLC. Therefore, for studying LC-derived EVs, we chose the cell lines NCI-H23 and A549 with an adenocarcinoma phenotype. The selected lung cell lines differed in their mutational status. The A549 cell line harbors an activating mutation in *KRAS* G12S and inactivation of the tumor suppressor *CDKN2A* gene, while NCI-H23 carries mutations in the *KRAS* (G12C) and *p53* genes [27]. The presence of a mutation in a *KRAS* gene determines resistance to EGFR tyrosine kinase inhibitor therapy [28].

To study CRC-derived EVs, we selected three colon cancer cell lines, Caco-2, HTC116, and HT29, which differ in their dysregulated pathways and their mutational status of the *KRAS* and *BRAF* genes. Caco-2 cells are microsatellite stable, CIN-positive (as in 70–90% of colorectal cancers), CIMP-positive, and harbor mutations in the *TP53* gene but not in the *BRAF* or *KRAS* gene. HT29 cells are microsatellite stable, CIN-positive (as in 70–90% of colorectal cancers), CIMP-positive, and harbor mutations in the *BRAF*, *PIK3CA,* and *TP53* genes. HTC116 cells are microsatellite unstable (MSI phenotype), CIN-negative (like 10–20% of colorectal cancers), CIMP-positive, and harbor mutations in the *KRAS* and *PIK3CA* genes [29]. The detection of KRAS and BRAF mutations is a crucial step for the prediction of a response to targeted therapy resistance [9]. This mutation is normally mutually exclusive.

We performed the high-resolution mass-spectrometric profiling of EVs and the whole cell lysate (WhL) followed by label-free quantification to determine the proteomic cargo of EVs and reveal EV proteomic core, or universal EV markers, as well as markers that can distinguish the tissue origin and tumor variants within the same type of cancer. We also attempted to elucidate the biological significance of these factors, especially the involvement of EV markers in cancer progression and metastasis. Finally, to verify putative EV protein markers, we applied the targeted mass-spectrometric method, i.e., selected reaction monitoring (SRM) with stable isotope-labeled peptides standards (SIS).

## 2. Results

### 2.1. Proteins Identified in Extracellular Vesicles Are Enriched in Exosomal Markers

**Figure 1:**
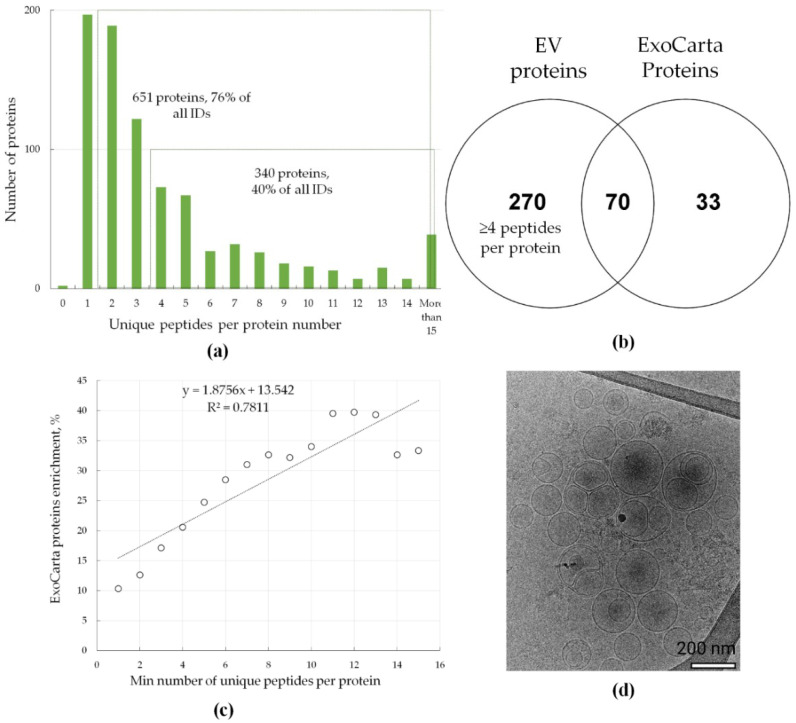
Mass-spectrometric analysis and Cryo-EM characterization of extracellular vesicles (EVs). (**a**) Distribution of proteins with different numbers of unique peptides per protein. Overall, 850 proteins were identified in all EV samples studied (potential contaminants, false positive identifications, and proteins identified only by peptides containing modifications were excluded). (**b**) Venn diagram that shows the intersection of proteins that were identified by at least 4 unique peptides (the most confidently identified proteins) and proteins that were often identified in exosomes (http://exocarta.org/exosome_markers_new, ExoCarta Top100 list); (**c**) correlation between enrichment in ExoCarta protein markers and peptide coverage of the identified proteins. ExoCarta protein enrichment is shown as the percentage of proteins often identified in exosomes (http://exocarta.org/exosome_markers_new, ExoCarta Top100 list) from the total number of identified proteins (y-axis). The peptide count of the identified proteins is shown as the minimal number of unique peptides per protein (x-axis); (**d**) Cryo-EM image of pooled EVs isolated from LC and CRC cells, bar size is 200 nm.

Mass spectrometric analysis of the EVs derived from lung cancer and colorectal cancer cells resulted in the identification of 850 proteins in all EV samples studied (potential contaminants, false positive identifications, and proteins identified only by peptides containing modifications were excluded). The mass-spectrometric data are available via ProteomeXchange with the identifiers PXD020467 and PXD020454. All proteins identified in the EV samples are listed in the Supplement 1. According to the guidelines for the interpretation of mass spectrometric data by the HPP (Human Proteome Project), the reliability of protein identification is increased if two or more unique peptides are mapped to the protein (https://www.hupo.org/HPP-Data-Interpretation-Guidelines). In general, the higher the coverage of the amino acid sequence of the protein, the more reliable the results obtained. Moreover, as shown previously, label-free quantification with several peptides per protein provides precise measurements and can detect even subtle biologically determined changes in the proteome, e.g., spaceflight-induced changes in mice liver [30]. Figure 1a represents the distribution of proteins with different numbers of unique peptides per protein.

Figure 1a shows that 651 proteins (76% of all identifications) were identified by at least two peptides, and 340 proteins (40% of all identifications) were identified by at least four peptides. The last protein subset was designated as the group with the most reliably identified proteins and used for label-free quantification. From the 103 proteins often identified in exosomes according to the ExoCarta database (ExoCarta Top100 list) (http://exocarta.org/exosome_markers_new), 70 proteins were overlapped with the most reliably identified protein subset. These included SDCB, TSG101, and ALIX, which are involved in exosome biogenesis, as well as integrins ITGA6 and ITGB1, MFGE8, and the lipid raft protein flotillin (FLOT1), which participate in cell adhesion. Additionally, protein abundance correlates with the protein peptide count and the number of unique peptides identified per protein [31]. Moreover, as Figure 1c shows, the most abundant proteins in the EV fraction belong to ExoCarta Top100 list (http://exocarta.org/exosome_markers_new). Conventional exosome markers, i.e., CD9, CD63, and CD81, have been also identified in mass-spectrometric experiments but only using one (CD63) or two (CD63 and CD81) unique peptides. The functional annotation analysis (Supplementary Figure S17) revealed 129 proteins that were annotated by GO terms “biological processes” as belonging to “vesicle-mediated transport” (FDR = 1 × 10^−46^), as well as 145 and 14 proteins that were annotated by GO terms “cellular component” (GO) as belonging to the “vesicle” group (FDR = 2.02 × 10^−44^) and “extracellular exosome” group (FDR = 4.87 × 10^−9^), respectively. Mass spectrometric analysis of the EV samples indicates that the proteins identified in EV samples are enriched in exosomal markers.

To assess the morphology and integrity of EVs, a pooled sample of EVs isolated from LC and CRC cells was visualized using cryo-electron microscopy (Cryo-EM) (Figure 1d). Typical round-shaped vesicular morphology was observed, with the diameter of the majority of EVs under 200 nm. This observation indicates successful isolation of small to medium size vesicles with an intact lipid bilayer.

### 2.2. Universal EV Proteins Distinctively Distinguish Whole Cell Lysate and EV Samples

**Figure 2:**
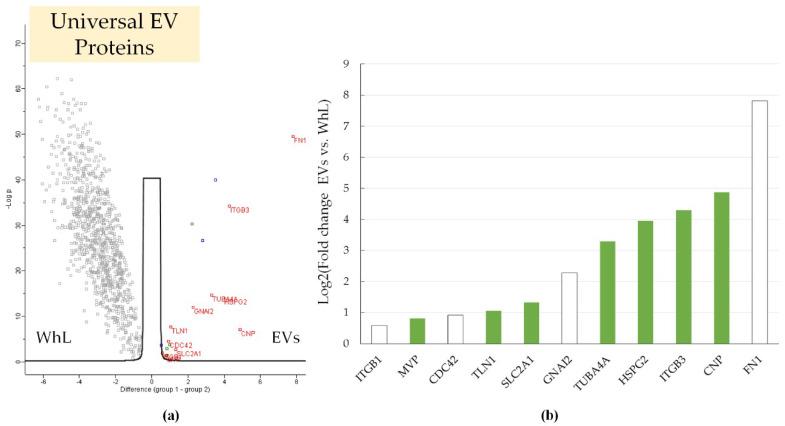
(**a**) Volcano plot that shows the differences in the protein abundance in the EV fraction (in red) and in the whole lysate (WhL) (in black) for all cell lines studied. A total of 933 proteins were identified by at least 4 unique peptides per protein and were statistically significant (Student’s *t*-test, truncation: permutation-based FDR = 0.01, S0 = 1, Perseus 1.6.0.7 software (Max Planck Institute of Biochemistry, Martinsried, Germany). EV markers (11 proteins) are shown by their gene names in red. Normalized data of label free quantification (LFQ) intensities were used for visualization of the protein level. The LFQ intensities were log2 transformed. Differences in protein abundance (considering the log2 transformation) are provided on the x-axis, while -Log *p* (log10 transformed *p*-value) is plotted on the y-axis. The blue dots represent proteins APOB, HBB, and HIST1H4A that could be artefacts of exosome isolation. Detailed data are presented in the Supplement 2; (**b**) universal EV protein markers were more abundant in the EV samples compared to WhL samples; the log2 transformed fold change that reflects the difference in protein abundance is plotted on the y-axis; proteins included in the ExoCarta Top100 list that contains the most often identified in exosome molecules (http://exocarta.org/exosome_markers_new) are shown in white.

Mass spectrometric analysis of EVs and WhL resulted in the identification of 3314 proteins (potential contaminants, false positive identifications, and proteins identified only by peptides containing modifications were excluded). Mass-spectrometric data are available via ProteomeXchange with the identifier PXD020454. To perform the relative protein quantification, 1859 proteins were used (identified using at least four peptides). Detailed results of the label-free quantification are presented in the Supplement 2. The result is shown in Figure 2. 

Two-sample testing (Student’s *t*-test, permutation-based false discovery rate (FDR) = 0.01) yielded 18 proteins (ANXA6, CDC42, CNP, FN1, GNAI2, HSPG2, ITGB1, ITGB3, JUP, MVP, RAP1B, SLC2A1, TLN1, TUBA4A, and UBE2N) that were significantly more abundant in the EV samples compared to the WhL samples. After two-sample testing, proteins with significant differences were subjected to additional filtering. First, the putative artefact of EV isolation, i.e., apolipoprotein B-100 (APOB) and hemoglobin subunit beta (HBB), were excluded from further analysis as they could be components of residual fetal bovine serum (FBS). Second, the peptides’ quality and their suitability for targeted analysis (see “Material and Method” section) were assessed. After filtration, 11 proteins remained (Figure 2b). Among them, the content of TUBA4A, HSPG2, ITGB3, CNP, and FN1 proteins in the EV samples was at least 10 times higher than that in WhL samples (Figure 2b). Proteins ITGB1, CDC42, GNAI2, and FN1 are often identified in exosomes (http://exocarta.org/exosome_markers_new, ExoCarta Top100 list). Seven proteins, i.e., MVP, TLN1, SLC2A1, TUBA4A, HSPG2, ITGB3, and CNP, may represent new markers of exosomes isolated from cells of epithelial origin. The revealed universal protein markers clearly distinguished the EVs from the WhL.

### 2.3. Lung Tissue-Specific EVs Loaded by EGFR Ligand EPS15 and Colon Tissue-Specific EVs Enriched in the Differentiation Regulator DMBT1

**Figure 3:**
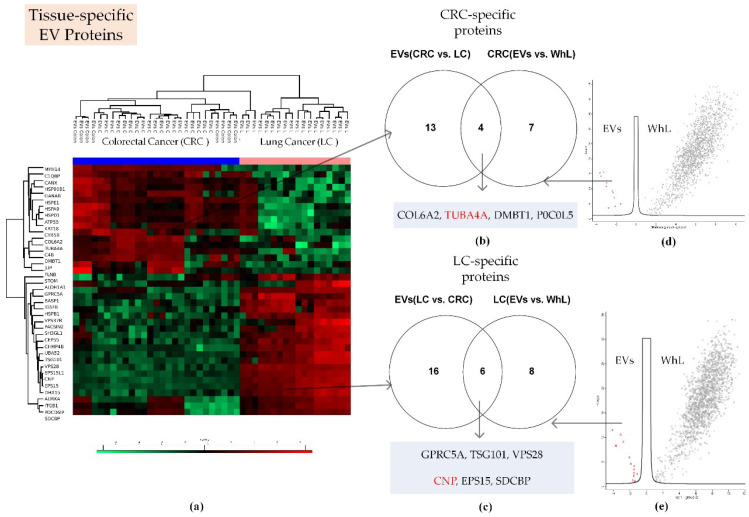
Proteomic search for tissue-specific EV proteins. (**a**) Heatmap that compares 39 proteins of EVs derived from lung cancer (LC) and colorectal cancer (CRC) cell lines (Figure S1 shows these proteins in detail); (**b**) Venn diagram that shows the intersection of proteins that were more abundant in CRC-derived EVs compared to LC-derived EVs, and proteins that were more abundant in EV samples compared to WhL samples of CRC cells; (**c**) Venn diagram that shows the intersection of proteins that were more abundant in LC-derived EVs compared to CRC-derived EVs and proteins that were more abundant in the EV samples compared to the WhL samples of LC cells; (**d**) volcano plot that shows the differences in protein abundance in the EV fraction (in red) and in the WhL (in black) for the CRC cell lines. Overall, 1654 proteins identified by at least 4 unique peptides per protein were statistically significant (Student’s *t*-test, truncation: permutation-based FDR = 0.01, S0 = 2, Perseus 1.6.0.7 software (Max Planck Institute of Biochemistry, Martinsried, Germany). Figure S2 shows the volcano plot in detail: (**e**) Volcano plot that shows the differences in protein abundance in the EV fraction (in red) and in the WhL (in black) for LC cell lines. Overall, 1622 proteins identified by at least 4 unique peptides per protein were statistically significant (Student’s *t*-test, truncation: permutation-based FDR = 0.01, S0 = 2, Perseus 1.6.0.7 software (Max Planck Institute of Biochemistry, Martinsried, Germany). Figure S3 shows the volcano plot in detail: For (**d**,**e**), normalized data of the LFQ intensities were used for visualization of the protein level. The LFQ intensities were log2 transformed. Differences in protein abundance (taking into account the log2 transformation) are shown on the x-axis, while -Log *p* (log10 transformed *p*-value) is plotted on the y-axis. Tissue-specific EV proteins that were also determined to be universal markers were highlighted in red. Detailed data are presented in the Supplement 3.

Label-free quantification and two-sample testing (Student’s *t*-test, FDR = 0.01) resulted in 39 proteins with significant differences in their abundance in lung tissue- and colon tissue-derived EVs (Figure 3).

Proteins with significant content differences were grouped into two clusters. These clusters comprised 17 and 22 proteins that were more abundant in CRC-derived EVs and LC-derived EVs, respectively (Figure 3; Supplement 3). Applying two-sample testing (Student’s *t*-test, FDR = 0.01), we determined 11 and 14 proteins to have levels at least twofold higher in the EV samples compared to the WhL samples of the CRC and LC cell lines, respectively (Supplement 3, Supplementary Figures S2 and S3). After overlapping the two sets of semi-quantitative data, 10 tissue-specific exosome markers were determined (Figure 3, Table 1). The TUBA4A and CNP proteins were also determined to be universal EV protein markers. SDCB, VPS28, and TSG101, as well as EGFR ligand EPS15, and retinol-inducible protein GPRC5A, which are involved in exosome biogenesis, were uniquely found to be specific for the lung tissue EV samples. Collagen COL6A2, the differentiation regulator DMBT1, and the complement component C4B (P0C0L5) were uniquely found to be specific for exosomes of the colon tissue.

### 2.4. Line-Specific EV Proteins Distinguish Different Variants within the Same Type of Cancer

LC cell lines A549 and NCI-H23, as well as CRC cell lines Caco-2, HTC116, and HT29, were used to search for line-specific EV proteins. The investigated cell lines carried mutations in the *KRAS*, *BRAF*, *PIK3CA*, *c-MYC*, and *P53* genes (Supplementary Table S1), which determine different susceptibility levels to pharmacological effects and various metastatic potentials.

**Figure 4:**
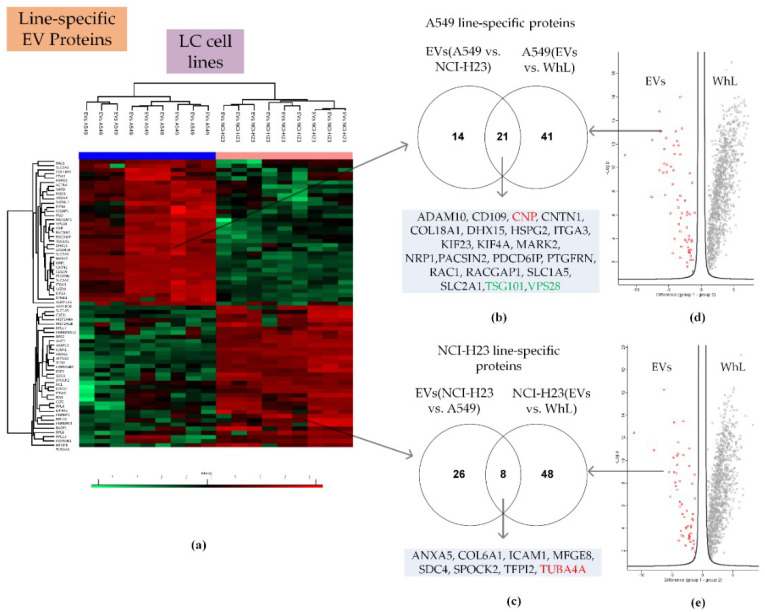
Proteomic search for LC line-specific EV proteins. (**a**) Heatmap that compares 69 proteins of EVs derived from the A549 and NCI-H23 cell lines (Figure S4 shows the heatmap in detail); (**b**) Venn diagram that shows the intersection of proteins that were more abundant in A549 line-derived EVs compared to NCI-H23 line-derived EVs and proteins that were more abundant in the EV fraction compared to the WhL of A549 cell line; (**c**) Venn diagram that shows the intersection of proteins that were more abundant in NCI-H23 line-derived EVs compared to A549 line-derived EVs and proteins that were more abundant in the EV fraction compared to the WhL of the NCI-H23 cell line; (**d**) volcano plot that shows the differences in protein abundance in the EV fraction (in red) and in the WhL (in black) for the A549 cell line. Overall, 1121 proteins identified by at least 4 unique peptides per protein were statistically significant (Student’s *t*-test, truncation: permutation-based FDR = 0.01, S0 = 2, Perseus 1.6.0.7 software (Max Planck Institute of Biochemistry, Martinsried, Germany). Figure S5 shows the volcano plot in detail; (**e**) the volcano plot that shows the differences in protein abundance in the EV fraction (in red) and in the WhL (in black) for the NCI-H23 cell line. Overall, 1173 proteins identified by at least 4 unique peptides per protein were statistically significant (Student’s *t*-test, truncation: permutation-based FDR = 0.01, S0 = 2, Perseus 1.6.0.7 software (Max Planck Institute of Biochemistry, Martinsried, Germany). Figure S6 shows the volcano plot in detail; for (**d**,**e**), normalized data of the LFQ intensities were used for visualization of the protein level. The LFQ intensities were log2 transformed. Differences in protein abundance (taking into account the log2 transformation) are plotted on the x-axis, while -Log *p* (log10 transformed *p*-value) is shown on the y-axis. Line-specific EV proteins that were also determined to be universal and tissue-specific markers are highlighted in red and green, respectively. Detailed data are presented in the Supplement 4.

Label-free quantification and two-sample testing (Student’s *t*-test, FDR = 0.01) resulted in 69 proteins with significant differences in abundance in A549 line-derived EVs and NCI-H23 line-derived EVs (Figure 4).

Proteins with significant content differences were grouped into two clusters. These clusters included 35 and 34 proteins that were more abundant in A549 line-derived EVs and NCI-H23 line-derived EVs, respectively. Comparison of the EVs and WhL proteomes revealed 62 and 56 proteins whose levels were at least twofold higher in the EV samples compared to the WhL samples of A549 and the NCI-H23 cell lines, respectively (Supplement 4, Supplementary Figures S5 and S6). The intersection of the two data sets yielded 21 and 8 proteins that were A549 line- and NCI-H23 line-specific, respectively (Figure 4, Table 1). The A549 line- and NCI-H23 line-specific protein subsets included the universal EV markers TUBA4A and CNP, as well as the tissue-specific EV markers TSG101 and VPS28.

**Figure 5:**
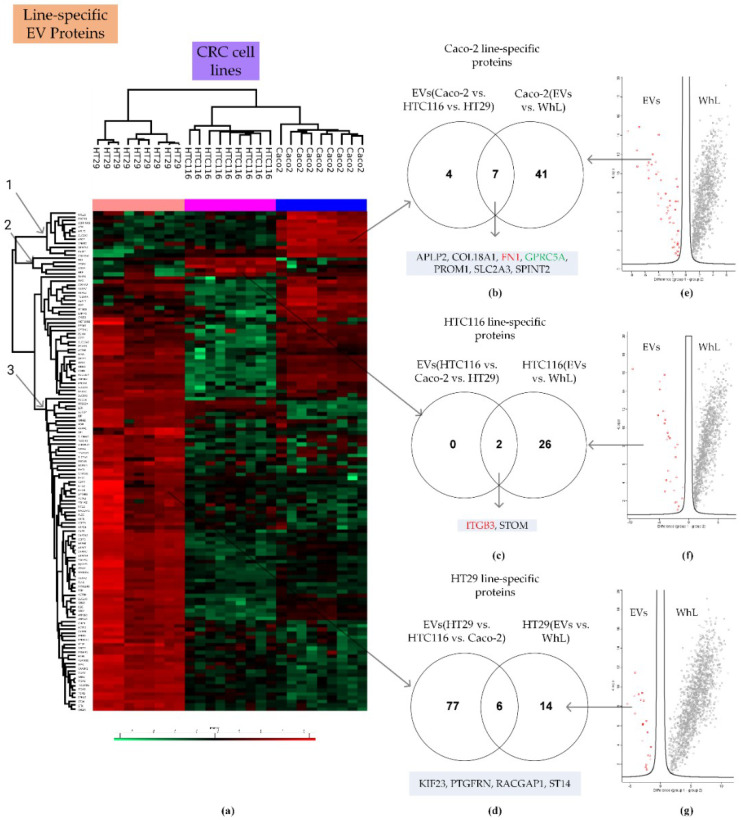
Proteomic search for CRC line-specific EV proteins. (**a**) Heatmap that compares the 132 proteins of EVs derived from the Caco-2, HTC116, and HT29 cell lines (Figure S7 shows the heatmap in detail); (**b**) Venn diagram that shows the intersection of protein that were more abundant in Caco-2 line-derived EVs compared to HTC116 and HT29 line-derived EVs and proteins that were more abundant in the EV fraction compared to the WhL of the Caco-2 cell line; (**c**) Venn diagram that shows the intersection of proteins that were more abundant in HTC116 line-derived EVs compared to Caco-2 and HT29 line-derived EVs and proteins that were more abundant in the EV fraction compared to the WhL of the HTC116 cell line; (**d**) Venn diagram that shows the intersection of proteins that were more abundant in HT29 line-derived EVs compared to HTC116 and Caco-2 line-derived EVs and proteins that were more abundant in the EV fraction compared to the WhL of HT29 cell line; (**e**) volcano plot that shows the differences in protein abundance in the EV fraction (in red) and in the WhL (in black) for the Caco-2 cell line. Overall, 1149 proteins identified by at least 4 unique peptides per protein were statistically significant (Student’s *t*-test, truncation: permutation-based FDR = 0.01, S0 = 2, Perseus 1.6.0.7 software (Max Planck Institute of Biochemistry, Martinsried, Germany). Figure S8 shows the volcano plot in detail; (**f**) the volcano plot that shows the differences in protein abundance in the EV fraction (in red) and in the WhL (in black) for the HTC116 cell line. Overall, 1241 proteins identified by at least 4 unique peptides per protein were statistically significant (Student’s *t*-test, truncation: permutation-based FDR = 0.01, S0 = 2, Perseus 1.6.0.7 software (Max Planck Institute of Biochemistry, Martinsried, Germany). Figure S9 shows the volcano plot in detail; (**g**) volcano plot that shows the differences in protein abundance in the EV fraction (in red) and in the WhL (in black) for the HT29 cell line. Overall, 1552 proteins identified by at least 4 unique peptides per protein were statistically significant (Student’s *t*-test, truncation: permutation-based FDR = 0.01, S0 = 2, Perseus 1.6.0.7 software (Max Planck Institute of Biochemistry, Martinsried, Germany). Figure S10 shows the volcano plot in detail; for (**e**–**g**) normalized data of the LFQ intensities were used for visualization at the protein level. The LFQ intensities were log2 transformed. Differences in protein abundance (taking into account the log2 transformation) are on the x-axis, while -Log *p* (log10 transformed *p*-value) is shown on the y-axis. Detailed data are presented in the Supplement 5.

Label-free quantification and multiple sample testing (ANOVA test, FDR = 0.01) resulted in 132 proteins with significant differences in abundances between Caco-2 line-derived EVs, HTC116 line-derived EVs, and HT29 line-derived EVs (Figure 5). Proteins with significant content differences were grouped into three clusters (Figure 5a). These clusters included 11 (cluster 1), two (cluster 2), and 86 (cluster 3) proteins that were more abundant in Caco-2 line-derived EVs, HTC116 line-derived EVs, and HT29 line-derived EVs, respectively. Two-sample testing of EVs and WhL proteomes yielded 48, 28, and 20 proteins, whose levels were at least twofold higher in the EV fraction compared to the WhL samples of the Caco-2, HTC116, and HT29 cell lines, respectively Supplement 5, Figures S8–S10). Pairwise intersection of the two data sets resulted in seven, two, and six proteins that were Caco-2 line-, HTC116 line-, and HT29 line-specific, respectively (Figure 5, Table 1). The Caco-2 line-, HTC116 line-, and HT29 line-specific protein subsets included the universal EV markers FN1 and ITGB3, as well as the tissue-specific EV marker, GPRC5A.

The universal, tissue-specific, and line-specific EV proteins are listed in Table 1. Their label-free results (fold change, *p*-value), annotations in terms of subcellular localization, and frequency of detection based on the “ExoCarta Top100 list” are presented in Table 2.

### 2.5. EV Protein Markers Are Enriched in Interactions and Are Involved in EGFR, Rap1, and Integrin Signaling

**Figure 6:**
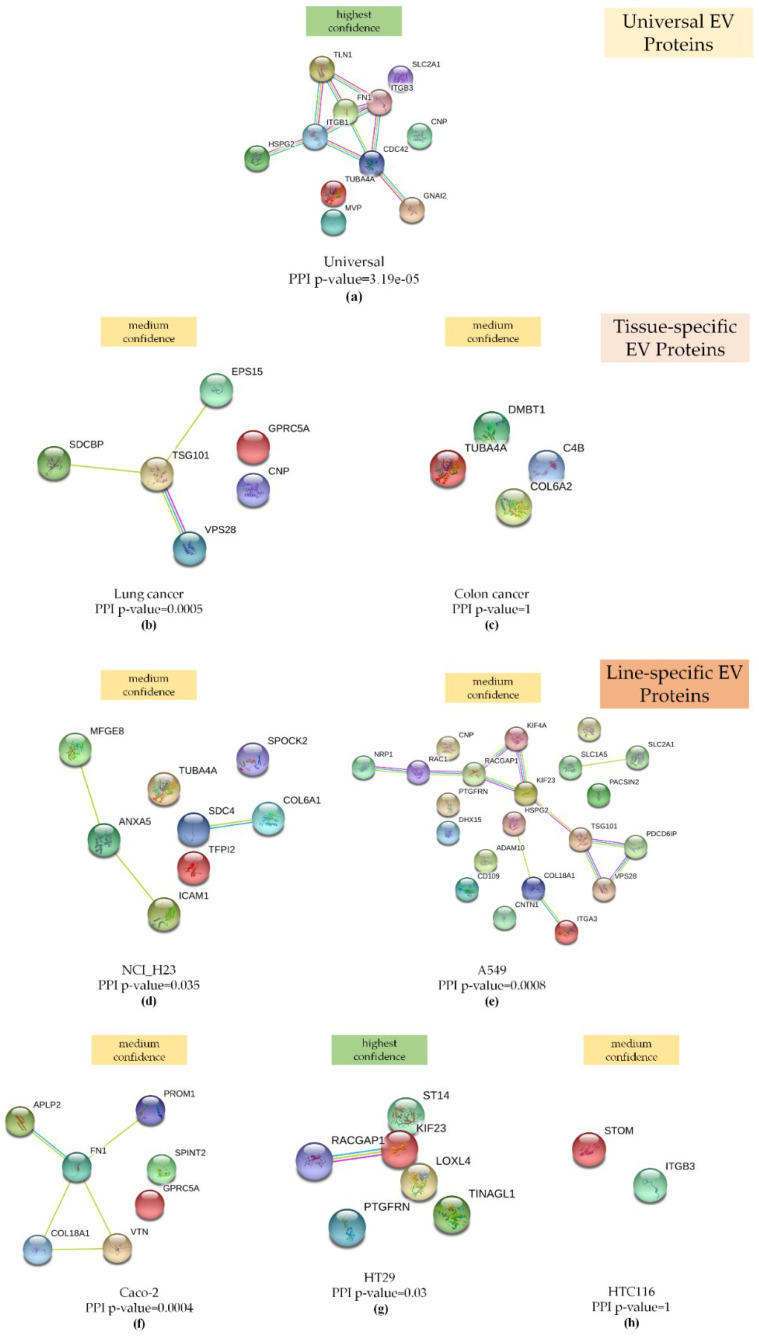
STRING interaction analysis (**a**) for 11 proteins that were more abundant in EVs compared to the WhL: The network was built based on the highest confidence (0.9) and enriched in interactions (PPI enrichment *p*-value = 3.19 × 10^−5^); (**b**) for the 6 proteins that were specific for lung tissue-derived EVs, the network was built based on medium confidence (0.4), and the network was enriched in interactions (PPI enrichment *p*-value = 0.0005); (**c**) for the 4 proteins that were specific for colon tissue-derived EVs, the network was built based on medium confidence (0.4), and the network was not enriched in interactions (PPI enrichment *p*-value = 1); (**d**) for the 8 proteins that were specific for NCI-H23-derived EVs, the network was built based on medium confidence (0.4), and the network was enriched in interactions (PPI enrichment *p*-value = 0.035); (**e**) for the 21 proteins that were specific for A549 cell-derived EVs, the network was built based on medium confidence (0.4), and the network was enriched in interactions (PPI enrichment *p*-value = 0.0008; (**f**) for the 7 proteins that were specific for Caco-2-derived EVs, the network was built based on medium confidence (0.4), and the network was enriched in interactions (PPI enrichment *p*-value = 0.035); (**g**) for the 6 proteins that were specific for HT29 cell-derived EVs, the network was built based on highest confidence (0.9), and the network was enriched in interactions (PPI enrichment *p*-value = 0.03); (**h**) for the 2 proteins that were specific for HTC116 cell-derived EVs, the network was built based on medium confidence (0.4), and the network was not enriched in interactions (PPI enrichment *p*-value = 1). All networks were enriched using the intersection of 8612 genes present on all platforms as the background, along with evidence from experimental protein–protein interactions (PPI) (purple lines), text-mining (bright green lines), and curated (turquoise blue lines) databases.

To assess the biological significance of universal, tissue-specific, and line-specific EV markers, we performed a STRING interaction analysis (Figure 6).

As expected, due to the small sample size, the colon tissue- and HTC116 cell-derived EV proteins were not enriched in their interactions (Figure 6a,h). At the same time, statistically significant interactions were established for lung tissue, NCI-H23 line-, A549 line-, and Caco-2 line-specific proteins with medium confidence, as well as for universal EV proteins and HT29 cell-derived EV proteins with the highest confidence.

The EV markers were also annotated based on their functions (Biological Processes (GeneOntology, GO), Kyoto Encyclopedia of Genes and Genomes (KEGG), Reactome Pathways) and subcellular localization (cellular component (GO)) where possible (Figures S11–S16).

Table 3 shows the statistically most confidant groups of biological functions, pathways, and subcellular localization annotations. The detailed data are presented in the Supplements 2–5.

The EV proteins of most groups were annotated as “extracellular exosome”, “extracellular region”, or “ESCRT I complex” by subcellular localization and belonged to the “extracellular matrix organization”, “vesicle-mediated transport”, “extracellular matrix organization”, “endosomal sorting complex required for transport (ESCRT)”, or “endocytosis” groups in terms of biological processes and pathways. These data support the EV identification. At the same time, EV proteins were involved in the “negative regulation of epidermal growth factor receptor signaling”, “Rap1 signaling pathway”, “microRNAs in cancer”, “integrin cell surface interactions”, “ECM-receptor interaction”, and “platelet activation, signaling, and aggregation” groups. Notably, most EV proteins within the annotation groups, even in the small-sized groups, were enriched in their interactions according to the STRING analysis. These results suggest that EVs regulate the oncogenic pathways, i.e., EGFR, Rap1, integrins, and MicroRNA signaling, and can contribute to metastasis and cancer progression in such a way.

### 2.6. Verification of Universal, Tissue-Specific, and Line-Specific EV Marker Levels by Targeted Mass-Spectrometry

**Figure 7:**
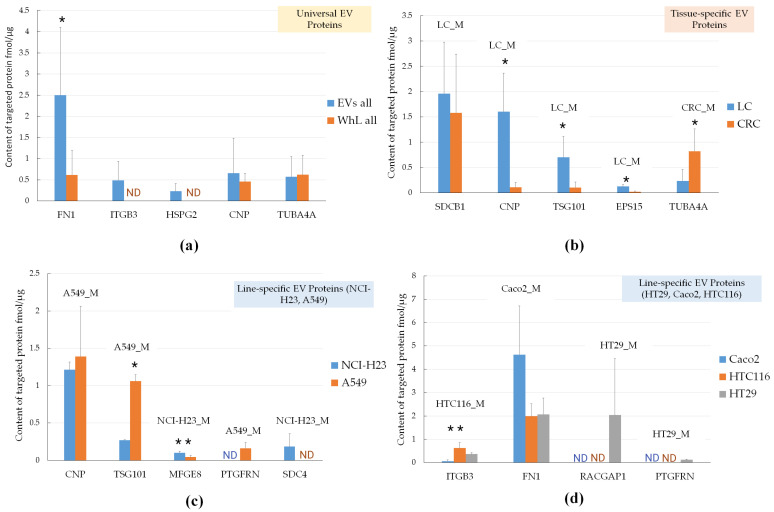
Selected reaction monitoring analysis (SRM) with the stable isotope-labeled peptides standards (SIS) analysis of EV proteins: (**a**) universal, (**b**) tissue-specific (LC_M and CRC_M), and (**c**) LC-line specific markers (NCI-H23_M, A549_M), and (**d**) CRC-line specific markers (HTC116_M, Caco2_M, and HT29_M). * *p*-value < 0.01; ** *p*-value < 0.05; Error bars represent standard deviation for measurements performed at 15, 9, 6, and 3 points for universal, lung tissue-specific, colon tissue-specific, and line-specific markers, respectively. For each cell line, EV isolation was performed in three biological replicates and yielded three EV sample. Each sample was analyzed using SRM with SIS in three technical replicates with coefficient of variation below 20%. ND—not detected.

To evaluate the levels of putative universal, tissue-specific, and line-specific EV markers, we applied SRM analysis with SIS. We measured the abundance of 12 proteins (CNP, EPS15, FN1, HSPG2, ITGB3, MFGE8, PTGFRN, RACGAP1, SDC4, SDCB1, TSG101, and TUBA4A) in all EV and WhL samples. Figure 7 demonstrates the levels of universal, tissue-specific, and line-specific EV markers.

Figure 7 shows that among the universal markers, ITGB3 and HSPG2 were detected in the EV samples only, and their measured levels were 0.48 ± 0.44 fmol/μg and 0.23 ± 0.18 fmol/μg, respectively. The FN1 protein content was fourfold (*p*-value = 1.1 × 10^−4^) higher in the EV samples compared to WhL. CNP and TUBA4A failed to be verified as universal markers, as there was no significant difference in their content. Nevertheless, three of five universal EV markers (FN1, ITGB3, and HSPG2) distinguished the EV samples from the WhL samples. For lung-specific markers, the CNP, TSGT101, and EPS15 content was 14.5-fold (*p*-value = 5.7 × 10^−5^), 6.6-fold (*p*-value = 7.7 × 10^−4^), and 5.9-fold (*p*-value = 3.3 × 10^−4^) higher in the LC-derived EVs compared to the CRC-derived EVs. The colon-specific marker TUBA4A was 3.6-fold (*p*-value = 3.7 × 10^−3^) more abundant in CRC-derived EVs compared to LC-derived EVs. The levels of the putative lung-specific marker SDCB1 were insignificantly higher in LC-derived EVs compared to CRC-derived EVs. Thus, four of five tissue-specific markers (CNP, TSG101, EPS15, and TUBA4A) distinguished LC-derived EVs from CRC-derived EVs. The A549-specific EV protein TSG101 levels were 3.4-fold (*p*-value = 2.9 × 10^−3^) higher in the A549 line-derived EV samples compared to the H23 line-derived EV samples. The A549-specific EV protein PTGFRN was detected only in the A549 line-derived EV samples at levels of 0.16 ± 0.07 fmol/μg. The NCI-H23-specific EV protein MFGE8 levels were 2.2-fold (*p*-value = 0.03) higher in NCI-H23 line-derived EV samples compared to the A549 line-derived EV samples. The NCI-H23-specific EV protein SDC4 was detected only in NCI-H23 line-derived EV samples at levels of 0.18 ± 0.17 fmol/μg. Therefore, four of five LC line-specific markers (TSG101, MFGE8, PTGFRN, and SDC4) distinguished NCI-H23 line-derived EVs from A549 line-derived EVs. The HT29 line-specific markers PTGFRN and RACGAP1 were detected in HT29-derived EV samples at levels of 0.12 ± 0.03 fmol/μg and 2.0 ± 2.4 fmol/μg, respectively. The HT116 line-specific marker ITGB3 content was significantly higher (ANOVA *p*-value = 0.01) in HT116-derived EVs compared to the other CRC cell lines. The Caco2 line-specific marker FN1 content was higher in Caco2-derived EVs compared to the other CRC cell lines, but the difference was not significant (ANOVA *p*-value = 0.08). Therefore, three out of four CRC line-specific markers (ITGB3, PTGFRN, and RACGAP1) distinguished HT29-derived, HTC116-derived, and Caco-derived EVs. The high standard deviation calculated for biological replicates can be the result of errors introduced by the sample preparation procedure, e.g., interfering with proteins of residual FBS that can contaminate EV proteins.

## 3. Discussion

At a qualitative level, the mass spectrometric analysis allowed us to determine the components of the ESCRT complex (TSG101 and PDCD6IP) and the proteins associated with exosome biogenesis (SDCBP, SDC4, VPS28, VPS37B, MFGE8, ARF6, VPS32, CD82, FLOT1, and FLOT2) [12,13], which were identified in our experiments with high confidence (by at least four unique peptides). Moreover, exosome-characteristic tetraspanins CD63 (one unique peptide), CD9 (two unique peptides), and CD81 (two unique peptides) were identified by means of shotgun mass-spectrometry, but they did not pass the four unique peptide threshold for label-free quantification. Thus, mass-spectrometric profiling allowed for the simultaneous analysis of exosome-characteristic proteins in the EV samples. At the same time, EVs are heterogeneous in their composition and traits [13]. Therefore, the results of the mass spectrometric analysis showed an average proteomic landscape of EVs. The enrichment of various EV populations followed by mass spectrometric analysis is required for a more accurate characterization of EV protein composition.

Our experimental dataset included proteins that were determined as LC and CRC markers: TSPAN1, LGALS3BP, SLC1A5, and GPRC5A were determined in HT29 and HCT116 line-derived EVs [23], YWHAZ was revealed in HTC116-derived vesicles [32], and ICAM1, PTGFRN, DMBT1, and FN1 were identified in NCI-H23-derived EVs [33], as well as the diagnostically valuable KRAS and EGFR.

At the same time, a number of proteins apparently originating from supplemental FBS (e.g., APOA1, APOB, fibrinogen chains, etc.) were identified. This observation suggests that residual FBS from the media contaminated the samples and interfered with the mass-spectrometric analysis as FBS-derived peptides compete with EV-derived peptides for MS acquisition. Due to the conservative amino acid sequences in mammals, the proteins from FBS could be identified as human EV proteins, thus masking valuable biological data. The fact that cultural media supplemented with 10–20% FBS can mimic diluted blood plasma will need to be taken into account when performing a proteomic analysis on plasma-isolated EVs. Highly abundant plasma proteins such as albumin, α-2-macroglobulin (A2M), and hemoglobin subunit α (HBA1) have been assigned to EVs isolated from blood plasma [34] and are listed among “ExoCarta Top100 list” (http://exocarta.org/exosome_markers_new) (i.e., albumin and A2M), but it is very difficult to confirm their EVs’ origins.

Using label-free mass-spectrometric profiling, we determined 11 proteins whose levels were higher in all EV samples compared to WhL. We denominated them as universal EV markers. Applying SRM with SIS, we verified FN1, ITGB3, and HSPG2 as EV-specific proteins. Previously, FN1 was determined to be an LC-associated marker on A549 cell line-derived and blood-derived EVs using mass-spectrometric profiling [25]. Moreover, FN1 was found to be up-regulated in the blood-derived EVs of smokers and patients with chronic obstructive pulmonary disease by proteomic methods [34]. Fibronectin is a ubiquitous and essential component of the extracellular matrix (ECM). It plays a role in tissue remodeling and wound-healing alongside the HSPG2 protein [35], which we also verified as a universal EV protein in our study. The verified universal EV marker ITGB3 serves as a receptor for components of the ECM, including FN1, and plays roles in the progression of different cancer-associated processes, including initiation, proliferation, survival, migration, and invasion [36]. Notably, we named the core EV proteins “universal EV proteins”, but the applicability of this term to other cell types (liver, brain, skin epithelium, etc.) must be proven. Nevertheless, six so-called universal EV proteins in our study (FN1, GNAI2, ITGB1, CDC42, MVP, and TLN1) overlapped with the core EV proteins determined from proteomic profiling of the EVs derived from 60 cell lines of different origins (NCI-60) [37].

For biological processes, some markers are associated with pro-cancerous properties and are involved in tumor progression and metastasis. According to the database annotation, universal EV proteins (CDC42, GNAI2, ITGB3, TLN1, and TUBA4A) are involved in platelet activation that help cancer cells escape immune surveillance and provide a prometastatic microenvironment [38]. These universal EV proteins, i.e., CDC42, GNAI2, ITGB1, ITGB3, and TLN1, were assigned to the Rap1 signaling pathway that regulates cell invasion and metastasis by affecting cell adhesion and modulating the expression of matrix metalloproteinases [39]. The Ras-associated protein-1 (Rap1) that triggers the Rap1 signaling pathway was identified in our study using eight peptides, and its abundance was at least four-fold higher in the EV fraction compared to the WhL samples.

Among the CRC-cell line markers, the Caco-2 line-specific EV protein Prominin-1 (PROM1), CD133, is a marker of cancer stem cells and associated with metastasis in CRC, and PROM1 overexpression renders tumors resistant to chemotherapy and radiation therapy [40]. The knock-down of APLP2, which is also a Caco-2 line-specific EV protein, reduced the proliferation of this particular cell line [41]. For LC-cell line markers, the A549 line-specific EV proteins CD109 and PTGFRN were found to be metastasis-associated in lung cancer [42]. Moreover, the CD109 protein triggered the process of metastasis in an NSCLC mice model against a similar genetic background of the human A549 cell line, including mutation in the *KRAS* gene [43]. The NCI-H23 line-specific EV protein ICAM-1 promotes cell–endothelial adhesion, which is an important step in metastasis development [44].

Several EV markers apparently involved in miRNA regulation could be oncogenic. The HT29 line-derived EV proteins ST14, and KIF23 were annotated against the KEGG database as belonging to the signaling pathway “MicroRNAs in cancer”. The proteins involved in RNA-mediated gene silencing, i.e., TSN, DHX9, SND1, MOV10, and CNOT1, were identified in our experiments by at least four peptides, but their levels were higher in the WhL samples compared to the EVs derived from the HT29 cell line.

These results highlight the oncogenic proteomes of EVs that are associated with LC and CRC progression and metastasis, the components of which represent a promising source of predictive and prognostic markers.

The verified lung cancer-specific EV protein EPS15 is of special interest. The EPS15 protein, i.e., the marker of the epidermal growth factor receptor substrate 15, is involved in the receptor-mediated endocytosis of EGFR [45]. Overexpression of the EPS15 gene is considered to be a favorable prognostic factor [46]. Moreover, in our study, EPS15 and the other lung cancer-specific EV markers, GPRC5 and TSG101, were associated with the “negative regulation of the epidermal growth factor receptor” signaling pathway. The EGFR itself was identified in both LC cell lines.

Furthermore, a number of EV markers play the role of tumor suppressors and inhibit proliferation. The CRC-specific EV marker DMBT1 may act as a tumor suppressor [34,35], whose loss could be a poor prognostic factor [47]. Specific for the CRC cell line HT29, the suppressor of tumorigenicity 14 (ST14) protein maintains epithelial barrier integrity and suppresses intestinal carcinogenesis [48]. The ability to suppress cancer metastasis was shown for HTC116 line-specific EV protein stomatin (STOM) [49]. The serine peptidase inhibitor Kunitz type 2 (SPINT2), a Caco-2 line-specific EV protein, inhibits HGF and suppresses the progression of various types of cancer [50]. The universal marker MVP was shown to assist in the removal of tumor suppressor microRNAs (miR-193a) from cancer cells [51]. These data also suggest that tumor suppressor proteins could be withdrawn from cancer cell by EVs.

The controversial cancer-related functions of EV proteins suggest that EV groups with specific molecular signatures and diversified functions may be isolated. Putatively, one EV subset may function for cancer cell as an oncosuppressive molecule disposal system, and the other EV subset may be a means for the transmission of an oncogenic signal. However, this concept must be experimentally proven.

The label-free quantitative analysis determined universal, tissue-specific, and line-specific EV protein markers. We verified 12 EV markers (CNP, EPS15, FN1, HSPG2, ITGB3, MFGE8, PTGFRN, RACGAP1, SDC4, SDCB1, TSG101, and TUBA4A) via targeted mass-spectrometry (SRM using SIS, 1 peptide per protein). These proteins could be the backbone for the development of an SRM-based assay for LC and CRC screening. The next step will be the validation of EV markers on plasma samples from patients with LC and CRC. In this case, putative LC and CRC biomarkers should be analyzed against the complex background of EVs originating from blood cells, i.e., platelets, erythrocytes, and leukocytes [52]. Previously, we studied EVs isolated from the blood plasma of healthy volunteers and used SRM analysis to determine the levels of the exosomal markers CD9, CD82, and HSPA8 [53]. According to the literature, the content of FIN1, TUBA4A, and MVP proteins, identified by us as universal EV markers, up-regulated in the blood plasma of patients with NSCLC compared to healthy donors [54].

Being prometastatic or tumor suppressive, the proteins in EVs play a crucial role in tumorigenesis and, therefore, have high diagnostic potential. The simultaneous targeted mass-spectrometric measurement of EV markers in human blood is a promising liquid biopsy tool.

## 4. Materials and Methods

To derive EVs, we used the colon cancer cell lines Caco-2, HT29, and HCT-116, as well as the lung cancer cell lines NCI-H23 and A549 as model objects. Cell culturing was performed in the presence of FBS to avoid starvation and oxidative stress. To prevent the contamination of the human EV proteome via bovine EV proteins, the culture medium was supplemented with exosome-depleted FBS prior to the mass-spectrometric analysis. The starting volume was rather small (18 mL) compared to that used in previous studies on EV isolation form cultural medium (120–500 mL [37,55]) to make the exosome isolation protocol applicable to liquid biopsy volume (7.5–40 mL [23,56,57]) in the future.

### 4.1. Cultivation of Cell Lines

The lung adenocarcinoma cell lines A549 and NCI-H23 and colorectal cancer cell lines HT29, HCT-116, and CaCo-2 were obtained from the cell culture bank Institute of Biomedical Chemistry (IBMC), Moscow, Russia.

For proteomic analysis of the lung adenocarcinoma cell lines (A549 and NCI-H23) and colorectal adenocarcinoma cell lines (HT29, HCT-116, and CaCo-2), the cell lines were cultured in a medium supplemented with exosome-depleted FBS.

Cell lines were cultured until reaching 70–80% confluency in an atmosphere of 5% CO_2_ at 37 °C, in a DMEM/F-12 medium without glutamine (PanEco, Moscow, Russia), with the addition of 10% FBS (in the case of Caco-2, the content of FBS was 20%) (Thermo Fisher Scientific, Waltham, MA, USA), 1% GlutaMAX (Thermo Fisher Scientific, Waltham, MA, USA), 1% essential amino acids (NEAA, Thermo Fisher Scientific, Waltham, MA, USA), and 1% antimycotic antibiotics (amphotericin B 0.25 μg/mL, penicillin G 100 units/mL, streptomycin 100 μg/mL). Microscope images of cell lines are shown in Supplementary Figure S18.

When the cells reached the monolayer (70–80% confluency), they were washed twice with potassium phosphate buffer (PBS), and the culture medium was replaced with an exosome-free medium (with the addition of FBS previously purified from exosomes via ultracentrifugation at 100,000× *g* for 14 h). For further analysis, the culture medium was collected after 24 h.

Cells were detached from the culture flasks via the addition of 2 mL of 0.25% trypsin-EDTA (Gibco™, Paisley, UK) for 5–10 min at 37 °C. The number of cells was measured using a cell counter and a cell viability analyzer—TC20 ™ Automated Cell Counter (BioRad, Hercules, CA, USA), as well as a cell counting kit (BioRad, Hercules, CA, USA). Cell viability ranged from 97.1% to 99.6% (Supplementary Figure S19). All cell lines were tested for their mycoplasma contaminations.

### 4.2. Isolation of EVs from the Culture Medium and Sample Preparation for Mass Spectrometry Analysis

Isolation of the exosomes from 18 mL of the culture medium supplemented with FBS and their tryptic digestion were carried out as described previously [53]. Briefly, the culture medium in an equal volume of 18 mL was centrifuged at 5000× *g* for 30 min at 4 °C (SX4750A type rolor, Beckman Coulter, Allegra X-15R Centrifuge, Indianapolis, IN, USA) to remove cell debris. The resulting supernatant was passed through a 0.22-μm filter. After that, EVs were sedimented using an Optima MAX-XP Ultracentrifuge and a TLA-55 rotor (Beckman Coulter, Indianapolis, IN, USA) at 100,000× *g* (k-factor 123) for 120 min at 4 °C. The sediment was then resuspended in 50 μL of 0.015% sodium cholate in 0.1 M PBS, pH 7.4, and stirred with vertical rotation on a Bio RS-24 mini-rotator (Biosan SIA, Riga, Latvia) for 30 min at room temperature followed by ultracentrifugation under the conditions described above. The sediment obtained after the second ultracentrifugation was dissolved in 50 μL of 0.1 M PBS, pH 7.4, and layered on a 26% sucrose solution in a PBS (*ρ* = 1.1082 g/mL), followed by ultracentrifugation at 120,000× *g* (k-factor 102) for 120 min at 4 °C. The sediment was resuspended in 50 μL of 0.1 M PBS, pH 7.4, and frozen at −80 °C for subsequent proteomic analysis. For each cell line, exosome isolation was performed in three replicates.

In each case, the entire EV sample was mixed with ProteaseMAX detergent (Promega, Fitchburg, WI, USA) compatible with mass spectrometry analysis (final concentration 0.12%). A one-step disulfide bond break (reduction and alkylation) was performed in a 50 mM triethylammonium bicarbonate buffer (TEAB) (Sigma-Aldrich, St. Louis, MO, USA) (pH 8.5) containing 0.2% ProteaseMAX (Promega, Fitchburg, WI, USA), 30 mM tris (2 carboxyethyl) phosphine (TCEP) (Thermo Fisher Scientific, Waltham, MA, USA), and 50 mM chloroacetamide (CAA) (Sigma-Aldrich, St. Louis, MO, USA) at 42 °C for 1 h. The reaction mixture was diluted 5 times with buffer for trypsinolysis containing 50 mM TEAB (pH 8.5), and ProteaseMAX (Promega, Fitchburg, WI, USA) was added to a final concentration of 0.1% along with the trypsin solution containing 1 μg of trypsin (Promega, Fitchburg, WI, USA), followed by incubation overnight at 37 °C. Hydrolysis was stopped by adding formic acid (Sigma-Aldrich, St. Louis, MO, USA) to a final concentration of 5%.

In the obtained samples, the peptide concentrations were determined by the colorimetric method using a Pierce™ Quantitative Colorimetric Peptide Assay kit (Pierce, Rockford, IL, USA) in accordance with the manufacturer’s recommendations. The peptides were dried and dissolved in 0.1% formic acid to a final concentration of 2 μg/μL. Based on these measurements, an equal quantity of total peptides, i.e., 2 µg, was compared for all samples via label-free mass-spectrometric profiling. The total peptide amount is presented in Supplementary Table S2.

### 4.3. Obtaining WhL

Cells were washed from the culture medium by centrifugation in a cold PBS at 1500× *g* for 5 min at 4 °C, and the procedure was repeated 3 times. Ten volumes of a lysis buffer containing 1% SDS (Sigma-Aldrich, St. Louis, MO, USA) in 0.1 M Tris Cl (pH 7.6) were added to the pellet, and the samples were sonicated with a Bandelin Sonopuls probe (“BANDELIN electronic GmbH & Co. KG”, Berlin, Germany) at 50% power for 5 min on ice. Then, the samples were centrifuged for 15 min at 14,000× *g* at 4 °C. The protein concentration was determined by the colorimetric method using a Pierce™ BCA Protein Assay Kit (Pierce, Rockford, IL, USA) in accordance with the manufacturer’s recommendations.

### 4.4. Tryptic Digestion of the WhL

Tryptic digestion of the proteins was carried out according to the FASP (Filter-Aided Sample Preparation) protocol [58] with some changes. Briefly, each sample in an amount of 100 μg was transferred to concentration filters with a cut-off of 10 kDa (Merck Millipore Limited, Tullagree, Ireland) by centrifugation at 11,000× *g* for 15 min at 20 °C. To break the disulfide bonds, each sample was incubated with 30 mM Tris TCEP (Thermo Fisher Scientific, Waltham, MA, USA) and 50 mM CAA (Sigma-Aldrich, St. Louis, MO, USA) at 42 °C for 1 h. Then, the samples were washed 3 times with a buffer containing 8M urea (Sigma-Aldrich, St. Louis, MO, USA) in 100 mM Tris Cl, pH 8.5, and washed twice with 50 mM TEAB, pH 8.5, by centrifugation at 11,000× *g* for 15 min at 20 °C. Then, 100 μL of a buffer containing 0.02% ProteaseMAX (Promega, Fitchburg, WI, USA) in 50 mM TEAB (Sigma-Aldrich, St. Louis, MO, USA), pH 8.5, and trypsin (Promega, Fitchburg, WI, USA) at a “trypsin to total protein” ratio of 1:50 was added. The samples were incubated overnight at 37 °C. After incubation, the peptides were eluted by centrifugation at 11,000× *g* for 15 min at 20 °C, and the filter was washed twice with 50 μL of 1% formic acid. In the obtained samples, the peptide concentration was determined by the colorimetric method using a Pierce™ Quantitative Colorimetric Peptide Assay kit (Pierce, Rockford, IL, USA) in accordance with the manufacturer’s recommendations. The peptides were dried and dissolved in 0.1% formic acid to a final concentration of 2 μg/μL.

### 4.5. Shotgun Mass Spectrometry

Tandem mass spectrometric analysis was performed for each sample in three technical replicates. The peptide mixture was loaded onto a Zorbax 300SB-C18 trap column (5 μm particle diameter, 5 × 0.3 mm) (Agilent Technologies, Santa Clara, CA, USA) and washed with the mobile phase C (5% acetonitrile in 0.1% formic acid and 0.05% trifluoroacetic acid) at a flow rate of 3 μL/min for 5 min. The peptides were separated on an analytical column Zorbax 300SB-C18 (3.5 μm particle diameter, 150 mm × 75 μm) (Agilent Technologies, Santa Clara, CA, USA) in a mobile phase B gradient (80% solution of acetonitrile in 0.1% formic acid) at a flow rate of 0.3 μL/min. The following parameters of the acetonitrile gradient were used: The analytical column was washed with a 2% mobile phase B for 3 min, and then the concentration of the mobile phase B was linearly increased to 40% for 67 min. Then, for 2 min, the concentration of the mobile phase B was increased to 100%, and the analytical column was washed for 9 min with 100% mobile phase B. Next, the concentration of the mobile phase B was reduced to 2% for 2 min, and the analytical column was balanced with 2% mobile phase B for 7 min.

Mass spectrometry analysis was performed using a Q Exactive™ HF Hybrid Quadrupole-Orbitrap™ Mass Spectrometer (Thermo Scientific, Waltham, MA, USA) equipped with an Orbitrap mass analyzer. Mass spectra were acquired in the positive ion mode with a resolution of 60,000 (*m/z* = 400) for the MS and 15,000 (*m/z* = 400) for the MS/MS scans. The AGC target was set to 3 × 10^6^ and 2 × 10^5^ with a maximum ion injection time of 25 and 150 ms for the MS and MS/MS levels, respectively. The survey MS scan was followed by the MS/MS spectra of the 20 most abundant precursors if the AGC target was greater than 10^4^. HCD fragmentation with the normalized collision energy (NCE) set to 28% was used. The dynamic exclusion duration was 60 s.

### 4.6. Data Analysis: Protein Identification and Label-Free Relative Quantitation

For identification and label-free quantification, mass spectrometry data were loaded into the MaxQuant software (version 1.6.0.16, Max Planck Institute of Biochemistry, Martinsried, Germany). Proteins were identified using the built-in Andromeda algorithm. Identification was carried out using the FASTA file (Uniprot release 25-10-2019, EMBL-EBI, Hinxton Cambridge, UK) and its inverted counterpart to calculate the frequency of false positive identifications (FDR), alongside a built-in database of potential contaminants. The carbamidomethylation of cysteine was used as a fixed modification, and methionine oxidation and N-terminal acetylation were used for variable modification. The tolerance for the precursor and fragment ions was 20 ppm. For proteins and peptides, the FDR threshold value was 0.01. Quantitative analysis was carried out on the basis of the area under the peak of the parent ion with calculation of the LFQ value performed using the algorithm built into MaxQuant (version 1.6.0.16, Max Planck Institute of Biochemistry, Martinsried, Germany) [59]. Unique peptides without modifications were used for the quantitative assessment. Potential contaminants, false positive identifications, and proteins identified only by peptides containing modifications were removed from the potentially identified proteins.

The statistical analysis was performed in the Perseus 1.6.0.7 software (Max Planck Institute of Biochemistry, Martinsried, Germany). To compare the 3 groups, we used a multi-sample ANOVA test. To compare the two groups, we used a two-sample *t*-test. The FDR threshold value of permutation (correction for multiple comparisons) was 0.01, S0 = 2. We compared the proteins for which at least 4 unique peptides per protein were identified.

Venn diagrams were generated using the online tool Venny version 2.1 (BioinfoGP, Madrid, Spain).

The STRING database v.11.0 was used to retrieve the protein–protein interactions (PPIs) from the lists of EV proteins. A medium (0.4) and high confidence (0.9) score were applied. The active interaction sources were text mining, experiments, and databases. The built-in functional enrichment analysis results according to the cellular components (GO), reactome pathways, and KEGG pathways (where available) were used for visualization.

### 4.7. Synthesis of SIS

The target peptides were selected from the shotgun mass-spectrometric data: Analysis of 5 cell lines in 3 biological replicates and in 3 technical replicates resulted in 45 LC-MS/MS runs. The criteria of selection were as follows: For universal markers, the LFQ intensity of the proteins had to be calculated in at least 40 of the 45 LC-MS/MS runs. The amino acid sequence had to be unique within the biological species *Homo sapiens.* The amino acid sequence did not contain cysteine (C), methionine (M), *N*-terminal glutamic acid (E), glutamine (Q), or tryptophan (W) and was missing hydrolysis sites. The length of peptides had to be within the range of 9–20 amino acid residues. For proteins with a large number of peptides, the quality of the high-resolution MS spectra was manually evaluated.

Solid-phase peptide synthesis was performed using the Overture™ Robotic Peptide Library Synthesizer (Protein Technologies, Manchester, UK), as described previously [60]. In the synthesis of isotope-labeled peptides, the isotopically-labeled amino acids Fmoc-Lys-OH-13C6.15N or Fmoc-Arg-OH-13C6.15N (Cambridge Isotope Laboratories, Cambridge, MA, USA) were used instead of the usual lysine or arginine.

### 4.8. Quantitative Analysis of EV Markers by Targeted Mass-Spectrometry

Each experimental sample was analyzed in three technical replicates. The measurements were carried out on the same samples as a shotgun spectrometric analysis. Before analysis, the samples were dried in a vacuum concentrator and reconstituted in 0.1% formic acid containing SIS in an equimolar concentration of 200 fmol/µL. The final content of each SIS was 40 fmol/ µg of total peptides.

Chromatographic separation was performed using an Agilent 1200 series system (Agilent Technologies, Santa Clara, CA, USA) connected to a TSQ Quantiva triple quadrupole mass analyzer (Thermo Scientific, Waltham, MA, USA). A sample was separated using an analytical column ZORBAX SB-C18 (150 × 0.5 mm, 5 μm particle diameter) (Agilent Technologies, Santa Clara, CA, USA) in a gradient of acetonitrile with a flow rate of 20 μL/min. First, the column was equilibrated with 5% solution B (80% acetonitrile in 0.1% formic acid) and 95% solution A (0.1% formic acid) for 5 min. Then, the concentration of solution B was linearly increased to 50% for 30 min, after which the concentration of solution B was increased to 99% in 1 min, and the column was washed with 99% solution B for 5 min. Then, the concentration was returned to the initial conditions for 1 min, in which the column was balanced for 9 min. A mass spectrometry analysis was performed in the dynamic selected-reaction monitoring (dSRM) mode using the following settings of the MS detector: The capillary voltage was 4000 V, the velocity of the drying gas (nitrogen) was 7 L/min, the velocity of the axillary gas (nitrogen) was 5 L/min, the capillary temperature was 350 °C, the isolation window for the first and third quadrupole was 0.7 Da, the scan cycle time was 1.2 s, and the collision gas (argon) pressure in the second quadrupole was set at 1.5 mTorr. The retention time window on the reverse phase column was 2.2 min for each precursor ion. The transition and normalized collision energy (V) lists are presented in the Supplement 6 (Sheet “SRM Table”). The data were loaded into the Skyline software v4.1.0 (MacCoss Lab Software, Seattle, WA, USA), where the SRM spectra were manually evaluated. The ratio of natural peptides to their SIS counterparts was automatically calculated for each peptide.

### 4.9. Cryo-EM

Prior to Cryo-EM study 3 μL of the sample were applied to Lacey Carbon EM grid treated with a glow discharge (30 s, 25 mA) in Pelco EasiGlow. After blotting for 2.5 s at 4 °C the grid with the specimen was plunge-frozen into a liquid ethane chilled with liquid nitrogen in Vitrobot Mark IV (Thermo Fisher Scientific, Waltham, MA, USA).

Cryo-EM study was carried out on a Titan Krios 60-300 (Thermo Fisher Scientific, Waltham, MA, USA) transmission electron microscope equipped with direct electron detector Falcon II (Thermo Fisher Scientific, Waltham, MA, USA) and a Cs image corrector (CEOS, Heidelberg, Germany) at accelerating voltage of 300 kV. Images were collected at 18kx magnification (pixel size 3.7 Å) in low-dose mode using EPU software (Thermo Fisher Scientific, Waltham, MA, USA).

## 5. Conclusions

Studying the proteomic cargo of EVs revealed the proteins associated with platelet activation, EGFR, Rap1, integrin, and microRNA signaling that could regulate metastasis and cancer progression. The EV protein subsets can distinguish different tissues and cell lines within the same type of cancer. Additionally, the proteomic core of the EVs of an epithelial lineage was established. The resulting EV protein list will provide a backbone for the development of a targeted mass-spectrometry assay that can be applied as a liquid biopsy tool.

## Figures and Tables

**Table 1:** Summary table for universal, tissue-specific, and line specific EV proteins.

EV Marker Type	Uniprot AN *	Gene Name	U	LC	CRC	A549	H23	HT29	Caco-2	HCT116
U	P60953	*CDC42*	+							
U	P04899	*GNAI2*	+							
U	Q14764	*MVP*	+							
U	P05106	*ITGB3*	+							+
U	P11166	*SLC2A1*	+			+				
U	P05556	*ITGB1*	+							
U	P68366	*TUBA4A*	+		+		+			
U	Q9Y490	*TLN1*	+							
U	P98160	*HSPG2*	+			+				
U	P09543	*CNP*	+	+		+				
U	P02751	*FN1*	+						+	
LC	Q8NFJ5	*GPRC5A*		+					+	
LC	Q99816	*TSG101*		+		+				
LC	Q9UK41	*VPS28*		+		+				
LC	P42566	*EPS15*		+						
LC	O00560	*SDCBP*		+						
CRC	P12110	*COL6A2*			+					
CRC	P0C0L5	*P0C0L5*			+					
CRC	Q9UGM3	*DMBT1*			+					
A549	P63000	*RAC1*				+				
A549	Q15758	*SLC1A5*				+				
A549	P39060	*COL18A1*				+			+	
A549	Q9H0H5	*RACGAP1*				+		+		
A549	O95239	*KIF4A*				+				
A549	Q8WUM4	*PDCD6IP*				+				
A549	Q9UNF0	*PACSIN2*				+				
A549	O43143	*DHX15*				+				
A549	Q7KZI7	*MARK2*				+				
A549	Q02241	*KIF23*				+		+		
A549	P26006	*ITGA3*				+				
A549	Q6YHK3	*CD109*				+				
A549	Q12860	*CNTN1*				+				
A549	Q9P2B2	*PTGFRN*				+		+		
A549	O14786	*NRP1*				+				
A549	O14672	*ADAM10*				+				
H23	P12109	*COL6A1*					+			
H23	Q08431	*MFGE8*					+			
H23	Q92563	*SPOCK2*					+			
H23	P31431	*SDC4*					+			
H23	P48307	*TFPI2*					+			
H23	P08758	*ANXA5*					+			
H23	P05362	*ICAM1*					+			
HT29	Q9Y5Y6	*ST14*						+		
Caco-2	O43490	*PROM1*							+	
Caco-2	Q06481	*APLP2*							+	
Caco-2	O43291	*SPINT2*							+	
Caco-2	P11169	*SLC2A3*							+	
HCT116	P27105	*STOM*								+

* AN—accession number.

**Table 2:** Label-free results (fold change, *p*-value); annotations in terms of subcellular localization (Uniprot database) and frequency of detection based on the “ExoCarta Top100 list” (http://exocarta.org/exosome_markers_new).

**Marker Type**	**Uniprot AN**	**Gene Name**	**FC EVs vs. WhL**	***p*-Value (*t*-test)**	Peptides Number *	ExoCarta Top100 **	Subcellular Localization ***
U	P02751	*FN1*	225	2.7 × 10^50^	57	45	Extracellular matrix, Secreted
U	P09543	*CNP*	29.3	8.3 × 10^−8^	20	-	Membrane
U	P05106	*ITGB3*	19.6	2.79 × 10^−5^	13	-	Plasma membrane
U	P98160	*HSPG2*	15.5	8.3 × 10^−15^	58	-	Extracellular matrix or secreted
U	P68366	*TUBA4A*	9.8	2.3 × 10^−15^	5	+	Cytoskeleton
U	P04899	*GNAI2*	4.8	1.1 × 10^−12^	5	53	Plasma membrane Cytoskeleton
U	P11166	*SLC2A1*	2.5	2.1 × 10^−3^	6	-	Plasma membrane
U	Q9Y490	*TLN1*	2.1	2.1 × 10^−8^	31	-	Plasma membrane Cytoskeleton
U	P60953	*CDC42*	1.9	2.8 × 10^−5^	4	56	Plasma membrane Cytoskeleton
U	Q14764	*MVP*	1.7	0.04	18	-	Cytoplasma, Nucleus
U	P05556	*ITGB1*	1.5	0.01	13	60	Plasma membrane, Endosome
			**FC EVs (LC vs. CRC)**	***p*** **-value (*t*-test)**			
LC	Q8NFJ5	*GPRC5A*	11.4	2.8 × 10^−13^	4	-	Plasma membrane
LC	Q99816	*TSG101*	24.2	4.1 × 10^−16^	13	76	Endosome
LC	Q9UK41	*VPS28*	15.7	1.8 × 10^−14^	5	-	Cell membrane, Endosome, Membrane
LC	P42566	*EPS15*	15.1	3.6 × 10^−27^	12	-	Endosome, Membrane
LC	O00560	*SDCBP*	2.9	8.9 × 10^−6^	6	78	Extracellular region or secreted
LC (U)	P09543	*CNP*	385	8.2 × 10^−27^	20	-	Membrane
			**FC EVs (CRC vs. LC)**	***p*** **-value (*t*-test)**			
CRC	P12110	*COL6A2*	3.6	2.5 × 10^−6^	7	-	Extracellular region or secreted
CRC	P0C0L5	*P0C0L5*	2.8	5.6 × 10^−8^	4	-	Extracellular region or secreted
CRC	Q9UGM3	*DMBT1*	15.7	1.7 × 10^−14^	4	-	Extracellular region or secreted
CRC(U)	P68366	*TUBA4A*	3.4	2.7 × 10^−6^	5	-	Cytoskeleton
			**FC EVs (A549 vs. H23)**	***p*** **-value (*t*-test)**			
A549	P63000	*RAC1*	2.3	4.2 × 10^−3^	5	53	Plasma membrane
A549	Q15758	*SLC1A5*	3.4	0.02	6	-	Plasma membrane
A549	P39060	*COL18A1*	5.7	5.6 × 10^−5^	8	-	Extracellular region or secreted
A549	Q9H0H5	*RACGAP1*	20.8	7.7 × 10^−5^	19	-	Plasma Membrane, nucleus
A549	O95239	*KIF4A*	3.0	9.6 × 10^−4^	14	-	Cytoskeleton, Nucleus
A549	Q8WUM4	*PDCD6IP*	3.0	1.3 × 10^−7^	26	96	Extracellular region or secreted
A549	Q9UNF0	*PACSIN2*	6.1	2.6 × 10^−7^	5	-	Cytoskeleton, Endosome, Membrane
A549	O43143	*DHX15*	5.1	1.0 × 10^−7^	26	-	Nucleus
A549	Q7KZI7	*MARK2*	4.8	3.5 × 10^−6^	4	-	Plasma Membrane, cytoskeleton
A549	Q02241	*KIF23*	25.8	4.0 × 10^−7^	28	-	Cytoskeleton, nucleus
A549	P26006	*ITGA3*	12.2	9.2 × 10^−11^	10	-	Plasma Membrane
A549	Q6YHK3	*CD109*	23.6	6.7 × 10^−14^	15	-	Plasma Membrane
A549	Q12860	*CNTN1*	94.2	1.3 × 10^−17^	21	-	Plasma Membrane
A549	Q9P2B2	*PTGFRN*	21.0	5.3 × 10^−10^	13	46	Endoplasmic reticulum, Golgi apparatus, Membrane
A549	O14786	*NRP1*	9.8	7.7 × 10^−11^	6	-	Plasma Membrane Extracellular region or secreted
A549	O14672	*ADAM10*	11.7	5.2 × 10^−7^	12	-	Plasma Membrane, Golgi apparatus
A549(U)	P11166	*SLC2A1*	3.5	0.01	6	-	Plasma membrane
A549 (U)	P98160	*HSPG2*	5.9	0.001	58	-	Extracellular matrix or secreted
A549 (LC, U)	P09543	*CNP*	4.0	7.3 × 10^−6^	20	-	Membrane
A549 (LC)	Q99816	*TSG101*	5.6	8.1 × 10^−6^	13	76	Endosome
A549 (LC)	Q9UK41	*VPS28*	3.9	2.3 × 10^−4^	5	-	Cell membrane, Endosome, Membrane
H23	P12109	*COL6A1*	3.1	9.2 × 10^−4^	7	-	Extracellular region or secreted
H23	Q08431	*MFGE8*	2.9	0.003	13	52	Extracellular region or secreted
H23	Q92563	*SPOCK2*	10.8	4.3 × 10^−8^	8	-	Extracellular region or secreted
H23	P31431	*SDC4*	6.1	4.6 × 10^−8^	4	-	Extracellular region or secreted
H23	P48307	*TFPI2*	8.5	3.9 × 10^−11^	4	-	Extracellular region or secreted
H23	P08758	*ANXA5*	2.5	1.0 × 10^−11^	12	67	Extracellular region or secreted, Plasma Membrane, Cytosol
H23	P05362	*ICAM1*	8.5	6.5 × 10^−10^	9	-	Extracellular region or secreted, Plasma Membrane
H23 (U)	P68366	*TUBA4A*	1.4	0.03	5	+	Cytoskeleton
			**FC EVs (HT29 vs. Caco-2 and HTC116)**	***p*** **-value (ANOVA)**			
HT29	Q9Y5Y6	*ST14*	6.5	6.5 × 10^−21^	9	-	Extracellular region or secreted, Plasma Membrane
HT29 (A549)	Q9H0H5	*RACGAP1*	50.8	5.2 × 10^−5^	19	-	Plasma Membrane, nucleus
HT29 (A549)	Q9P2B2	*PTGFRN*	4.9	6.6 × 10^−11^	13	46	Endoplasmic reticulum, Golgi apparatus, Membrane
HT29 (A549)	Q02241	*KIF23*	57.1	1.9 × 10^−6^	28	-	Cytoskeleton, nucleus
			**FC EVs (Caco-2 vs. HT29 and HTC116)**	***p*** **-value (ANOVA)**			
Caco-2	O43490	*PROM1 (CD133)*	3.4	1.0 × 10^−6^	5	-	Plasma Membrane
Caco-2	Q06481	*APLP2*	43.5	9.2 × 10^−17^	13	-	Plasma Membrane, Nucleus
Caco-2	O43291	*SPINT2*	7.9	7.5 × 10^−10^	5	-	Extracellular region or secreted, Plasma Membrane
Caco-2	P11169	*SLC2A3*	41.3	2.8 × 10^−18^	5	-	Plasma Membrane
Caco-2 (A549)	P39060	*COL18A1*	3.6	2.6 × 10^−9^	8	-	Extracellular region or secreted
Caco-2 (LC)	Q8NFJ5	*GPRC5A*	9.2	2.5 × 10^−6^	4	-	Plasma membrane
Caco-2 (U)	P02751	*FN1*	2.6	7.1 × 10^−8^	57	45	Extracellular matrix, Secreted
			**FC EVs (HTC116 vs. HT29 and Caco-2)**	***p*** **-value (ANOVA)**			
HCT116	P27105	*STOM*	3.1	9.1 × 10^−6^	7	44	Plasma Membrane, cytoskeleton
HTC116 (U)	P05106	*ITGB3*	3.1	3.2 × 10^−10^	13	-	Plasma membrane

* Number of peptides identified in EV samples only; ** frequency of detection based on the “ExoCarta Top100 list” (http://exocarta.org/exosome_markers_new) containing molecules commonly identified in exosomes; *** subcellular localization based on the Uniprot database.

**Table 3:** Summary of EV protein markers annotated against their Biological Processes (GO), KEGG Pathways, Reactome Pathways, and Cellular Components (GO).

Specific Group	N *	Biological Processes (GO)	Cellular Components (GO)	KEGG Pathways	Reactome Pathways
NCI-H23	8	NA	Extracellular region (ANXA5|ICAM1|MFGE8 **, SDC4, SPOCK2, TFPI2, TUBA4A, COL6A1,), FDR = 6.07 × 10^−6^	ECM-receptor interaction (**COL6A1|SDC4**), FDR = 0.0132	Extracellular matrix organization (**COL6A1|SDC4**, ICAM1), FDR = 0.0227
A549	21	Cellular component organization **NRP1|RAC1|RACGAP1|KIF23|KIF4A|PDCD6IP|TSG101|VPS28**, **HSPG2|COL18A1|ITGA3** (ADAM10, CNP, CNTN1, MARK2, PACSIN2, PTGFRN, SLC2A1), FDR = 2.78 × 10^−5^	Flemming body (**RACGAP1|KIF23|KIF4A|PDCD6IP|TSG101**, SLC2A1), FDR = 5.11 × 10^−6^	NA	Vesicle-mediated transport (**RAC1|RACGAP1|KIF23|KIF4A|TSG101|VPS28**, PACSIN2), FDR = 0.00046
HTC116	2	NA	Melanosome (ITGB3, STOM), FDR = 0.0029	NA	NA
Caco-2	7	Extracellular matrix organization (**COL18A1|FN1|VTN**, SPINT2), FDR = 8.8 × 10^−4^	Extracellular exosome (**FN1|PROM1**), FDR = 0.0057	NA	Integrin cell surface interactions (**COL18A1|VTN**), FDR = 0.0123
HT29	6	Actomyosin contractile ring assembly (**KIF23|RACGAP1**), FDR = 1.7 × 10^−4^	Central spindlin complex (**KIF23|RACGAP1**), FDR = 6.42 × 10^−5^	MicroRNAs in cancer (KIF23, ST14), FDR = 8.7 × 10^−4^	Kinesins (**KIF23|RACGAP1**), FDR = 0.0036
Lung	6	Negative regulation of epidermal growth factor receptor signaling (**EPS15|TSG101**, GPRC5A), FDR = 9.26 × 10^−5^	ESCRT I complex (**TSG101|VPS28**), FDR = 3.5 × 10^−4^	Endocytosis (**EPS15|TSG101|VPS28**) FDR = 3.77 × 10^−5^	ESCRT, (**TSG101|VPS28**), FDR = 2.9 × 10^−4^
Colon	4	NA	Extracellular region (C4B, COL6A2, DMBT1, TUBA4A), FDR = 0.02	NA	NA
Universal	11	Vesicle-mediated transport (**CDC42|FN1|HSPG2|ITGB1|ITGB3|TLN1**, MVP, TUBA4A), FDR = 4.2 × 10^−4^	Melanosome (**ITGB1|ITGB3**, CNP, SLC2A1), FDR = 6.43 × 10^−5^	Rap1 signaling pathway (**CDC42|GNAI2|ITGB1|ITGB3|TLN1**), FDR = 4.75 × 10^−6^	Platelet activation, signaling and aggregation (**CDC42|GNAI2|ITGB3|TLN1**, TUBA4A), FDR = 3.65 × 10^−5^

* EV protein total number: ** Proteins enriched in interactions according to the STRING interaction analysis are shown in bold and delimited by bars. NA: not available.

## Supplementary Materials

Supplementary materials can be found at https://www.mdpi.com/1422-0067/21/18/6601/s1. Supplement 1: EV_proteins_all_identified.xlsx; Supplement 2: Universal_EV_markers.xlsx; Supplement 3: Lung_and_Colon_EV_markers.xlsx; Supplement 4: NCI_H23_and_A549_EV_markers.xlsx; Supplement 5: Caco2_HT29_HTC116_EV_markers.xlsx; Supplement 6: SRM experiment.xlsx; Table S1: The characteristics of lung cancer and colorectal cancer cell lines; Table S2: Total peptide quantity obtained by Pierce™ Quantitative Colorimetric Peptide Assay kit after tryptic digestion; Figure S1: Heatmap comparing 39 proteins of EVs derived from lung cancer and colon cancer cell lines; Figure S2: Volcano plot shows the differences in proteins abundance in EV fraction (in red) and in whole lysate (WhL) (in black) for cell lines of colorectal adenocarcinoma; Figure S3: Volcano plot shows the differences in proteins abundance in EV fraction (in red) and in whole lysate (WhL) (in black) for cell lines of lung adenocarcinoma; Figure S4: Heatmap comparing 69 EV proteins derived from NCI-H23 and A549 cell lines; Figure S5: Volcano plot shows the differences in proteins abundance in EV fraction (in red) and in whole lysate (WhL) (in black) for A549 cell line of lung adenocarcinoma; Figure S6: Volcano plot shows the differences in proteins abundance in EV fraction (in red) and in whole lysate (WhL) (in black) for NCI-H23 cell line of lung adenocarcinoma; Figure S7: Heatmap comparing 132 EV proteins derived from HT29, HTC-116 and Caco-2 cell lines; Figure S8: Volcano plot shows the differences in proteins abundance in EV fraction (in red) and in whole lysate (WhL) (in black) for Caco-2 cell line of colorectal adenocarcinoma; Figure S9: Volcano plot shows the differences in proteins abundance in EV fraction (in red) and in whole lysate (WhL) (in black) for HTC-116 cell line of colorectal adenocarcinoma; Figure S10: Volcano plot shows the differences in proteins abundance in EV fraction (in red) and in whole lysate (WhL) (in black) for HT29 cell line of colorectal adenocarcinoma; Figure S11: Functional enrichments in NCI-H23-derived EV proteins set (8 proteins) according (a) GeneOntology (Cellular component); (b) KEGG Pathways and (c) Reactome Pathways; Figure S12: Functional enrichments in A549-derived EV proteins set (21 proteins) according (a) GeneOntology (Cellular component); (b) Reactome Pathways (c) GeneOntology (Biological process); Figure S13: Functional enrichments in Caco-2-derived EV proteins set (7 proteins) according (a) GeneOntology (Cellular component) (b) GeneOntology (Biological process; Figure S14: Functional enrichments in HT29-derived EV proteins set (6 proteins) according (a) GeneOntology (Cellular component); (b) Reactome Pathways.; (c) GeneOntology (Biological process); Figure S15: Functional enrichments in lung-derived EV proteins set (6 proteins) according (a) GeneOntology (Cellular component); (b) Reactome Pathways.; (c) GeneOntology (Biological process); Figure S16: Functional enrichments in universal EV proteins set (11 proteins) according (a) GeneOntology (Cellular component); (b) KEGG Pathways.; (b) Reactome Pathways.; (c) GeneOntology (Biological process); Figure S17: The STRING interaction analysis for 340 proteins; Figure S18: Microscope images of cell lines of LC (A549 (a) and NCI-H23 (b)) and CRC (Caco2 (c), HT29 (d), and HTC116 (e)); Figure S19: Cell viability results.

## Abbreviations

CAA	Chloroacetamide extracellular matrix
CIMP	Pathway characterised by the CpG island methylation phenotype
CIN	Chromosomal instability pathway
CRC	Colorectal cancer
CTCs	Circulating tumor cells
ctDNA	Circulating tumor DNA
ECM	Extracellular matrix
ESCRT	Endosomal sorting complex required for transport
EVs	Extracellular vesicles
FBS	Fetal bovine serum
FDR	False discovery rate
FISH	Fluorescence in situ hybridization
GO	GeneOntology,
IHC	Immunohistochemistry
ILVs	Intraluminal vesicles
KEGG	Kyoto Encyclopedia of Genes and Genomes
LC	Lung cancer
LC–MS/MS	Liquid chromatography-tandem mass spectrometry
LDCT	Low-dose computed tomography
LFQ	Label free quantification
miRNA	Micro RNA
MSI	Microsatellite instability
MVEs	Multivesicular endosomes
NSCLC	Non-small cell lung carcinoma
SIS	Stable isotope-labeled peptides standards
SRM	Selected reaction monitoring
TCEP	Tris(2 carboxyethyl)phosphine
TEAB	Triethylammonium bicarbonate buffer
WhL	Whole cell lysate
