# Supplementary material for: Proteomic Approach for Searching for Universal, Tissue-Specific, and Line-Specific Markers of Extracellular Vesicles in Lung and Colorectal Adenocarcinoma Cell Lines
Source: Int J Mol Sci. 2020 Sep 9;21(18):6601. doi: 10.3390/ijms21186601 (PMC7555231; doi:10.3390/ijms21186601)
Supplement: Supplementary file 1 [file ijms-21-06601-s001.zip › Supplementary materials.doc]

Supplementary Materials

**Table S1.** The characteristics of lung cancer and colorectal cancer cell lines.

| **Cell line** | **Mutation** | **Type of tumor** | **Ref.** |
| --- | --- | --- | --- |
| Caco-2 | *TP53* | primary tumor (сolorectal carcinoma) | [1] |
| HT-29 | *BRAF*, *PIK3CA* and *TP53* | primary tumor (сolorectal adenocarcinoma) | [1] |
| HTC116 | *KRAS*, and *PIK3CA* | primary tumor (сolorectal carcinoma) | [1] |
| A549 | activating mutation in *KRAS* G12S and inactivation of the tumor suppressor *CDKN2A* gene | primary tumor (NSCLC, adenocarcinoma) | [2] |
| NCI-H23 | KRAS-G12C, p53 | primary tumor  (NSCLC, adenocarcinoma) | [3] |

**Table S2.** Total peptide quantity obtained by Pierce™ Quantitative Colorimetric Peptide Assay kit after tryptic digestion.

| **Cell line** | **Replicate of EV isolation** | **Total peptide quantity in EV sample, μg** | **Total peptide quantity in WhL samples, µg *** | **Total protein quantity in WhL samples, µg**** | **Total protein Fold change WhL vs. EVs***** | **millions of cells** |
| --- | --- | --- | --- | --- | --- | --- |
| Caco-2 | 1 | 45.3 | 90.6 | 689.9 | 15.2 | 1 |
|  | 2 | 49.6 | 87.5 | 252.8 | 5.1 | 1 |
|  | 3 | 36.7 | 73.4 | 336.8 | 9.2 | 1 |
| HT-29 | 1 | 42.4 | 71.3 | 380.0 | 9.0 | 6 |
|  | 2 | 52.3 | 83.7 | 285.9 | 5.5 | 1.85 |
|  | 3 | 52.3 | 75.7 | 412.0 | 7.9 | 6 |
| HTC116 | 1 | 46.3 | 91.3 | 484.5 | 10.5 | 3.6 |
|  | 2 | 53.0 | 84.9 | 374.7 | 7.1 | 3.6 |
|  | 3 | 49.5 | 95.5 | 616.8 | 12.5 | 3.6 |
| A549 | 1 | 44.7 | 89.1 | 328.8 | 7.4 | 3 |
|  | 2 | 48.0 | 65.0 | 350.9 | 7.3 | 3 |
|  | 3 | 50.1 | 86.5 | 411.2 | 8.2 | 3 |
| NCI-H23 | 1 | 49.5 | 94.7 | 532.3 | 10.8 | 3 |
|  | 2 | 60.5 | 87.7 | 356.0 | 5.9 | 3 |
|  | 3 | 68.5 | 95.9 | 651.5 | 9.5 | 3 |

*derived from 100 μg of total protein measured by Pierce™ BCA Protein Assay Kit (Pierce, USA); ** derived from particular number of cells;***For EV samples total protein quantity considered equal to total peptide quantity [4].

**Figure S1.** Heatmap comparing 39 proteins of EVs derived from lung cancer and colon cancer cell lines.

WhL

EVs

**Figure S2.** Volcano plot shows the differences in proteins abundance in EV fraction (in red) and in whole lysate (WhL) (in black) for cell lines of colorectal adenocarcinoma. Overall 1654 proteins identified by at least 4 unique peptides per protein, statistically significant (Student’s t-test, truncation: permutation-based FDR=0.01, S0=2, Perseus 1.6.0.7 software (Max Planck Institute of Biochemistry, Germany). Colon-derived EV markers (4 proteins) is shown by its gene name in red. Normalized data of LFQ intensities were used for visualization in protein level. The LFQ intensities were log2 transformed. Differences in protein abundance (taking into account the log2 transformation) is on x-axis, -Log p (log10 transformed p-value) is on y-axis. Detailed data are represented in supplementary file Lung_and_Colon_EV_markers.xlsx.

EVs

WhL

**Figure S3.** Volcano plot shows the differences in proteins abundance in EV fraction (in red) and in whole lysate (WhL) (in black) for cell lines of lung adenocarcinoma. Overall 1622 proteins identified by at least 4 unique peptides per protein, statistically significant (Student’s t-test, truncation: permutation-based FDR=0.01, S0=2, Perseus 1.6.0.7 software (Max Planck Institute of Biochemistry, Germany). Lung-derived EV markers (6 proteins) is shown by its gene name in red. Normalized data of LFQ intensities were used for visualization in protein level. The LFQ intensities were log2 transformed. Differences in protein abundance (taking into account the log2 transformation) is on x-axis, -Log p (log10 transformed p-value) is on y-axis. Detailed data are represented in supplementary file Lung_and_Colon_EV_markers.xlsx.

**Figure S4.** Heatmap comparing 69 EV proteins derived from NCI-H23 and A549 cell lines.

WhL

EVs

**Figure S5.** Volcano plot shows the differences in proteins abundance in EV fraction (in red) and in whole lysate (WhL) (in black) for A549 cell line of lung adenocarcinoma. Overall 1121 proteins identified by at least 4 unique peptides per protein, statistically significant (Student’s t-test, truncation: permutation-based FDR=0.01, S0=2, Perseus 1.6.0.7 software (Max Planck Institute of Biochemistry, Germany). A549-derived EV markers (21 proteins) is shown by its gene name in red. Normalized data of LFQ intensities were used for visualization in protein level. The LFQ intensities were log2 transformed. Differences in protein abundance (taking into account the log2 transformation) is on x-axis, -Log p (log10 transformed p-value) is on y-axis. Detailed data are represented in supplementary file NCI_H23_and_A549_EV_markers.xlsx.

WhL

EVs

**Figure S6.** Volcano plot shows the differences in proteins abundance in EV fraction (in red) and in whole lysate (WhL) (in black) for NCI-H23 cell line of lung adenocarcinoma. Overall 1173 proteins identified by at least 4 unique peptides per protein, statistically significant (Student’s t-test, truncation: permutation-based FDR=0.01, S0=2, Perseus 1.6.0.7 software (Max Planck Institute of Biochemistry, Germany). NCI-H23-derived EV markers (8 proteins) is shown by its gene name in red. Normalized data of LFQ intensities were used for visualization in protein level. The LFQ intensities were log2 transformed. Differences in protein abundance (taking into account the log2 transformation) is on x-axis, -Log p (log10 transformed p-value) is on y-axis. Detailed data are represented in supplementary file NCI_H23_and_A549_EV_markers.xlsx.

**Figure S7.** Heatmap comparing 132 EV proteins derived from HT-29, HTC-116 and Caco-2 cell lines.

WhL

EVs

**Figure S8.** Volcano plot shows the differences in proteins abundance in EV fraction (in red) and in whole lysate (WhL) (in black) for Caco-2 cell line of colorectal adenocarcinoma. Overall 1149 proteins identified by at least 4 unique peptides per protein, statistically significant (Student’s t-test, truncation: permutation-based FDR=0.01, S0=2, Perseus 1.6.0.7 software (Max Planck Institute of Biochemistry, Germany). Caco-2-derived EV markers (7 proteins) is shown by its gene name in red. Normalized data of LFQ intensities were used for visualization in protein level. The LFQ intensities were log2 transformed. Differences in protein abundance (taking into account the log2 transformation) is on x-axis, -Log p (log10 transformed p-value) is on y-axis. Detailed data are represented in supplementary file Caco2_HT29_HTC116_EV_markers.xlsx.

WhL

EVs

**Figure S9.** Volcano plot shows the differences in proteins abundance in EV fraction (in red) and in whole lysate (WhL) (in black) for HTC-116 cell line of colorectal adenocarcinoma. Overall 1241 proteins identified by at least 4 unique peptides per protein, statistically significant (Student’s t-test, truncation: permutation-based FDR=0.01, S0=2, Perseus 1.6.0.7 software (Max Planck Institute of Biochemistry, Germany). HTC116-derived EV markers (2 proteins) is shown by its gene name in red. Normalized data of LFQ intensities were used for visualization in protein level. The LFQ intensities were log2 transformed. Differences in protein abundance (taking into account the log2 transformation) is on x-axis, -Log p (log10 transformed p-value) is on y-axis. Detailed data are represented in supplementary file Caco2_HT29_HTC116_EV_markers.xlsx.

WhL

EVs

**Figure S10.** Volcano plot shows the differences in proteins abundance in EV fraction (in red) and in whole lysate (WhL) (in black) for HT29 cell line of colorectal adenocarcinoma. Overall 1552 proteins identified by at least 4 unique peptides per protein, statistically significant (Student’s t-test, truncation: permutation-based FDR=0.01, S0=2, Perseus 1.6.0.7 software (Max Planck Institute of Biochemistry, Germany). HT29-derived EV markers (6 proteins) is shown by its gene name in red. Normalized data of LFQ intensities were used for visualization in protein level. The LFQ intensities were log2 transformed. Differences in protein abundance (taking into account the log2 transformation) is on x-axis, -Log p (log10 transformed p-value) is on y-axis. Detailed data are represented in supplementary file Caco2_HT29_HTC116_EV_markers.xlsx.

**Figure S11.** Functional enrichments in NCI-H23-derived EV proteins set (8 proteins) according (a) GeneOntology (Cellular component); (b) KEGG Pathways and (c) Reactome Pathways. The darker color indicates more statistical significance (FDR threshold<0.05). Detailed data are represented in supplementary file NCI_H23_and_A549_EV_markers.xlsx.

**Figure S12.** Functional enrichments in A549-derived EV proteins set (21 proteins) according (a) GeneOntology (Cellular component); (b) Reactome Pathways (c) GeneOntology (Biological process). The darker color indicates more statistical significance (FDR threshold<0.001). Detailed results are represented in supplementary file NCI_H23_and_A549_EV_markers.xlsx.

**Figure S13.** Functional enrichments in Caco-2-derived EV proteins set (7 proteins) according (a) GeneOntology (Cellular component) (b) GeneOntology (Biological process); the darker color indicates more statistical significance (FDR threshold<0.05). Detailed results are represented in supplementary file Caco2_HTC116_HT29_EV_markers.xlsx.

**Figure S14.** Functional enrichments in HT29-derived EV proteins set (6 proteins) according (a) GeneOntology (Cellular component); (b) Reactome Pathways.; (c) GeneOntology (Biological process). The darker color indicates more statistical significance (FDR threshold<0.05). Detailed results are represented in supplementary file Caco2_HTC116_HT29_EV_markers.xlsx.

**Figure S15.** Functional enrichments in lung-derived EV proteins set (6 proteins) according (a) GeneOntology (Cellular component); (b) Reactome Pathways.; (c) GeneOntology (Biological process). * shown only results FDR threshold<0.01. The darker color indicates more statistical significance (FDR threshold<0.05). Detailed results are represented in supplementary file Lung_and_Colon_EV_markers.xlsx.

**Figure S16.** Functional enrichments in universal EV proteins set (11 proteins) according (a) GeneOntology (Cellular component); (b) KEGG Pathways.; (b) Reactome Pathways.; (c) GeneOntology (Biological process); The darker color indicates more statistical significance (FDR threshold<0.01, shown groups with at least 3 components). Detailed results are represented in supplementary file Universal_EV_markers.xlsx.

**Figure S17.** The STRING interaction analysis for 340 proteins, that were identified at least by 4 unique peptides in EV samples of all cell lines studied: the network was built based on highest confidence (0.9), the network is enriched in interactions (PPI enrichment p-value= 1×10^-16^); evidence from experimental protein‐protein interaction; Green nodes represent 129 proteins that have been annotated by Biological Process (GO) as belonging to vesicle-mediated transport (FDR = 1×10^-46^). Red and blue nodes shows 145 and 14 proteins that have been annotated by Cellular Component (GO) as belonging to “Vesicle” group (FDR = 2.02×10^-44^) and “Extracellular exosome” group (FDR = 4.87×10^-9^), respectively.

**Figure S18.** Microscope images of cell lines of LC (A549 (a) and NCI-H23 (b)) and CRC (Caco2 (c), HT29 (d), and HTC116 (e)).

**Figure S19.** Cell viability results. The number of cells was measured using a cell counter and a cell viability analyzer - TC20 ™ Automated Cell Counter (BioRad) and a cell counting kit (BioRad). Averaged from 3 replicates is shown.

References

1.	Ahmed, D.; Eide, P. W.; Eilertsen, I. A.; Danielsen, S. A.; Eknæs, M.; Hektoen, M.; Lind, G. E.; Lothe, R. A. Epigenetic and genetic features of 24 colon cancer cell lines. *Oncogenesis* **2013**, *2*, e71, doi:10.1038/oncsis.2013.35.

2.	Korrodi-Gregório, L.; Soto-Cerrato, V.; Vitorino, R.; Fardilha, M.; Pérez-Tomás, R. From proteomic analysis to potential therapeutic targets: Functional profile of two lung cancer cell lines, A549 and SW900, widely studied in pre-clinical research. *PLoS One* **2016**, *11*, doi:10.1371/journal.pone.0165973.

3.	Blanco, R.; Iwakawa, R.; Tang, M.; Kohno, T.; Angulo, B.; Pio, R.; Montuenga, L. M.; Minna, J. D.; Yokota, J.; Sanchez-Cespedes, M. A gene-alteration profile of human lung cancer cell lines. *Hum. Mutat.* **2009**, *30*, 1199–1206, doi:10.1002/humu.21028.

4.	Zhang, B.; Pirmoradian, M.; Zubarev, R.; Kall, L. Covariation of peptide abundances accurately reflects protein concentration differences. *Mol. Cell. Proteomics* **2017**, *16*, 936–948, doi:10.1074/mcp.O117.067728.
